# Therapeutic potential of short-chain fatty acids for acute lung injury: a systematic review and meta-analysis of preclinical animal studies

**DOI:** 10.3389/fnut.2024.1528200

**Published:** 2025-01-08

**Authors:** Liying Xie, Linyan Wang, Yongxin Liao, Miaoen Yao, Tong Mai, Rongrong Fan, Yun Han, Gengbiao Zhou

**Affiliations:** ^1^The Second Clinical College of Guangzhou University of Chinese Medicine, Guangzhou, China; ^2^Guangdong Provincial Hospital of Chinese Medicine, Guangzhou, China; ^3^The Second Affiliated Hospital of Guangzhou University of Chinese Medicine, Guangzhou, China

**Keywords:** acute lung injury, meta-analysis, preclinical evidence, short-chain fatty acids, the gut-lung axis

## Abstract

**Background:**

Short-chain fatty acids (SCFAs), derived from the fermentation of dietary fiber by intestinal commensal bacteria, have demonstrated protective effects against acute lung injury (ALI) in animal models. However, the findings have shown variability across different studies. It is necessary to conduct a comprehensive evaluation of the efficacy of these treatments and their consistency.

**Objective:**

This systematic review and meta-analysis aimed to explore the effects of SCFAs on ALI based on preclinical research evidence, in order to provide new treatment strategies for ALI.

**Methods:**

We included studies that tested the effects of SCFAs on ALI in animal models. This study was performed according to the Preferred Reporting Items for Systematic Reviews and Meta-Analyses (PRISMA) guidelines. A comprehensive search for relevant studies was conducted in the PubMed, Embase, Web of Science, Cochrane Library, and China National Knowledge Infrastructure (CNKI) databases up to February 2024. The data were extracted in accordance with the established selection criteria, and the risk of bias was evaluated for each study.

**Results:**

A total of 16 articles were finally included in the meta-analysis. The results indicated that the SCFAs significantly reduced lung wet-to-dry weight (SMD = −2.75, 95% CI = −3.46 to −2.03, *p* < 0.00001), lung injury scores (SMD = −5.07, 95% CI = −6.25 to −3.89, *p* < 0.00001), myeloperoxidase (SMD = −3.37, 95% CI = −4.05 to −2.70, *p* < 0.00001), tumor necrosis factor-alpha (SMD = −3.31, 95% CI = −4.45 to −2.16, *p* < 0.00001) and malondialdehyde (SMD = −3.91, 95% CI = −5.37 to −2.44, *p* < 0.00001) levels in animal models of ALI. The results of the subgroup analysis indicated that the efficacy of SCFAs varies significantly with dosage and duration of treatment.

**Conclusion:**

SCFAs can reduce inflammation and oxidative stress in animal models of ALI. The clinical efficacy of SCFAs for ALI deserves further in-depth research.

**Systematic review registration:**

https://www.crd.york.ac.uk/PROSPERO/display_record.php?RecordID=584008, CRD42024584008.

## 1 Introduction

In recent years, with the outbreak of novel epidemic respiratory viruses, the deaths caused by the acute lung injury (ALI) has significantly increased, posing a substantial threat to human health (1, 2). ALI is a serious disease characterized by excessive inflammatory responses, usually triggered by various direct or indirect factors, including pneumonia, sepsis, trauma, and blood transfusions (3, 4). During the acute exudative phase of ALI, a large number of immune cells accumulate at the site of injury, initiating a series of inflammatory signaling pathways and releasing a range of pro-inflammatory cytokines (5). Subsequently, the inflammation disrupts the barrier function of the alveolar epithelium and endothelium, leading to increased permeability of the alveolar-capillary membrane (6). As a consequence of this persistent acute inflammatory process, the majority of patients with ALI will rapidly experience a deterioration in their respiratory function, which will eventually progress to the more severe form of and acute respiratory distress syndrome (ARDS) (7). Despite some advancements in the scientific understanding of the pathophysiological mechanisms underlying ALI, current treatment approaches still mainly depend on supportive therapies, including mechanical ventilation (8). ALI/ARDS remains one of the most fatal clinical syndromes in intensive care (4, 9). In light of these developments, researchers engaged in both clinical and preclinical studies are directing their attention toward the creation of novel treatments and medications, with the objective of providing patients with ALI/ARDS more efficacious therapeutic alternatives.

The emerging theory of the gut-lung axis elucidates a robust interconnection between the gut and the lungs (10). The gut microbiota and their metabolites have been demonstrated to play a role in the pathogenesis of various lung diseases, including asthma and respiratory infections, by regulating both innate and adaptive immunity (11). A substantial body of research indicates that the release of short-chain fatty acids (SCFAs) represents a pivotal mechanism through which the gut microbiota maintains host health and gut homeostasis (12). SCFAs are a type of fatty acid produced by beneficial bacteria in the gut through the fermentation of dietary fiber (13). In the intestines, the primary SCFAs include acetate, propionate, and butyrate (12). SCFAs can be absorbed and utilized by intestinal epithelial cells, with a portion serving as an energy source for cellular metabolic activities, while another portion enters the peripheral circulation to function as signaling molecules that regulate the host’s biological responses (14). They play an important role in regulating gut microbiota balance, maintaining intestinal barrier function, suppressing inflammation, and improving immune system function (15). The relationship between elevated levels of SCFAs in the gut and improved lung health is becoming increasingly clear (16). In patients with impaired immune function, an elevated concentration of SCFAs in their feces is associated with a markedly reduced risk of developing lung infections (14, 17). Currently, researchers have begun to explore the effects of exogenous supplementation of SCFAs in the treatment of ALI in animal experiments. For example, some studies have found that supplementing with SCFAs can significantly reduce the lung injury scores, lung wet-to-dry (W/D) ratio, myeloperoxidase (MPO), tumor necrosis factor-alpha (TNF-*α*), and malondialdehyde (MDA) levels in animal models of ALI (18–20). However, other studies have reached contradictory conclusions (21, 22). Therefore, it is necessary to summarize the results of the relevant publications to more comprehensively assess the impact of SCFAs on ALI.

Although there is currently an absence of clinical evidence regarding the application of SCFAs in patients with ALI/ARDS, the results of preclinical studies in animal experiments are invaluable for clinical practice and provide a crucial foundation for in-depth research into disease pathology and mechanisms (23). Our study aimed to evaluate the efficacy and potential mechanisms of SCFAs in ALI/ARDS animal models through a systematic review and meta-analysis. The findings could provide robust support for future experimental design and clinical application.

## 2 Materials and methods

### 2.1 Search strategy

A search in databases including PubMed, Cochrane Library, Embase, Web of Science, and China National Knowledge Infrastructure was performed from inception to February 2024.

The keywords of the search were as follows: “actue lung injury,” “acute respiratory distress syndrome,” “ALI,” “ARDS,” “short chain fatty acid,” “SCFA,” “acetate,” “propionate,” “butyrate.” After manual screening, additional studies were identified from relevant reviews and citations (the detailed search strategy is shown in Supplementary Table S1).

### 2.2 Inclusion and exclusion criteria

Studies included in this review must meet the following criteria: (1) the participants were rat or mouse animal models of ALI/ARDS; (2) the intervention drugs were solutions of SCFAs such as sodium acetate, sodium propionate or sodium butyrate, while the control group could be either a blank control or an equal volume of PBS or normal saline; and (3) the literature was published in Chinese and English. The exclusion criteria were as follows: (1) reviews, clinical trials, case reports, and protocols; (2) articles for which the full text was not available.

### 2.3 Data extraction

Two researchers independently reviewed the literature, extracted data and cross-checked the data, and the third researcher negotiated and adjudicated in case of disagreement. The main content of the data extraction included: first author’s name, year of publication, sample size of each group, animal species, modeling methods, duration, types and dose of SCFAs, and outcome-related indicators. For articles that only reported data in the form of images, we used Origin 2022 software to extract relevant data from the images. When administering different doses or durations of SCFAs, we recorded all data meticulously for subsequent subgroup analysis. The primary outcome measure was the lung W/D ratio and lung injury scores, while the secondary outcome measures included MPO, TNF-*α*, and MDA.

### 2.4 Quality assessment

The SYRCLE’s risk of bias tool was used to assess the risk of bias in the included studies (24). It was developed on the basis of the Cochrane risk of bias tool and consisted of 10 entries. The results were assessed using “yes,” “no” and “unclear” to represent low, high and unclear risk of bias, respectively (25).

### 2.5 Statistical analyses

The analysis was conducted using RevMan 5.2 (Cochrane Collaboration, Oxford, United Kingdom) and Stata 14.0 (StataCorp, TX, United States) software. Considering that the results of the outcome indicators were all continuous variables, the standardized mean difference (SMD) was calculated with 95% confidence interval (CI) as the overall effects. Heterogeneity was assessed using I^2^, and a value exceeding 50% was considered to indicate high heterogeneity. For each outcome measure, specified a random-effects model of analysis. Subgroup and sensitivity analyses were performed to explore potential heterogeneity between studies and to identify different sources of confounding. The presence of publication bias was identified through the use of funnel plots and Egger’s test. Finally, we employed trim-and-fill methods to detect potential asymmetry and to assess the robustness of the conclusions.

## 3 Results

### 3.1 Selection and characteristics of included studies

**Figure 1:**
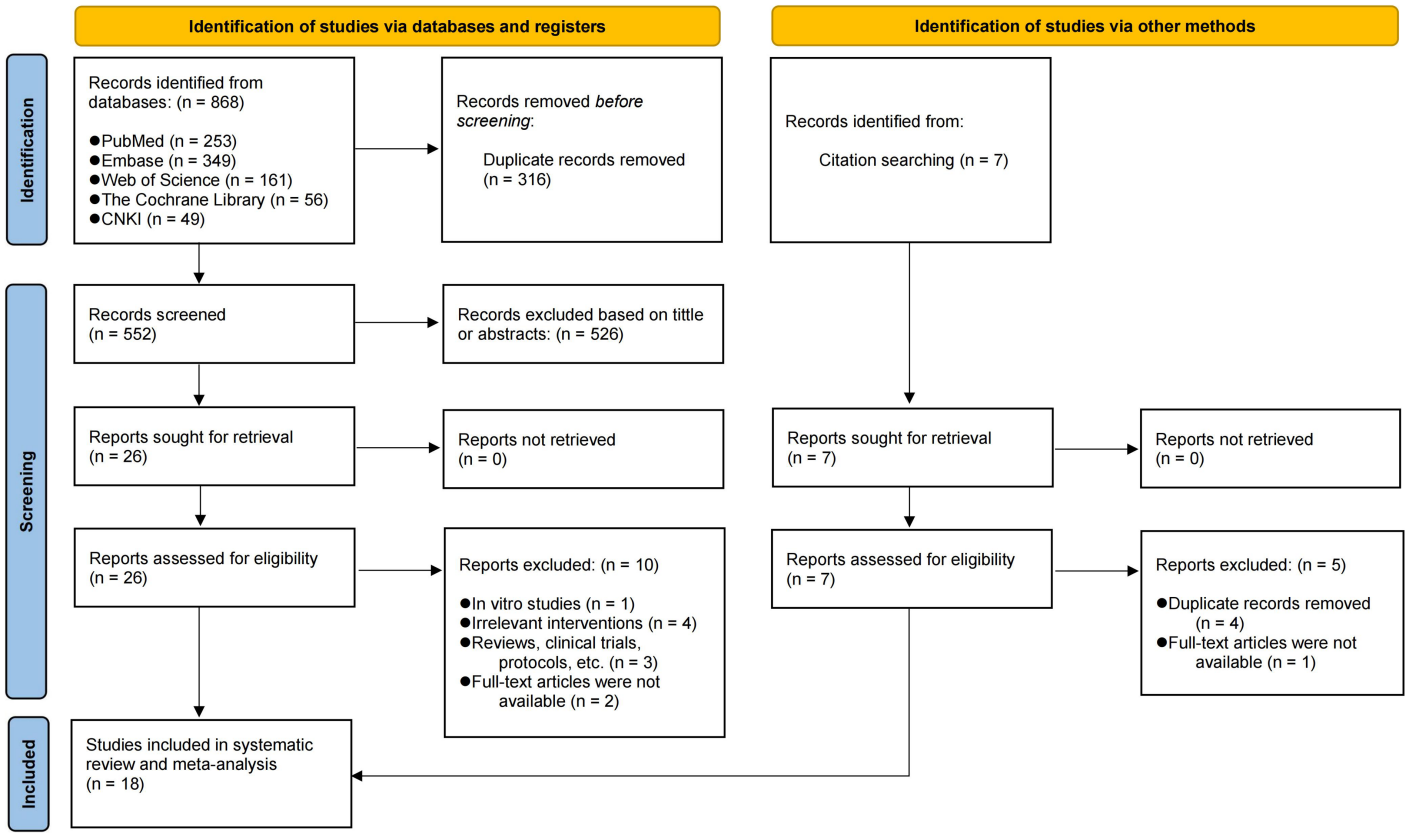
PRISMA flow diagram of the study selection process.

As shown in Figure 1, we initially obtained 868 articles, of which 316 were identified as duplicates and subsequently excluded. In addition, seven articles were identified through citation searches. According to the predetermined screening criteria, 18 eligible articles were finally included (18–21, 26–39). All studies described the species of experimental animals, with 10 articles using mice and the remainder using SD rats. The most frequently utilized modeling methods in all articles were intratracheal injection of lipopolysaccharide (LPS) and cecal ligation and puncture (CLP) to induce ALI. A total of 11 articles used sodium butyrate as the intervention, while three articles employed acetate, two articles used a solution of mixed SCFAs, one used propionate, and one used both acetate and propionate simultaneously. A detailed account of the specific details and characteristics of each included study can be found in Table 1 and Supplementary Table S2.

**Table 1:** Main characteristics of the 18 included studies.

Author (Year)	Animal species (Gender)	Model	Sample size (EG/CG)	Intervention			
				Type of SCFAs	Method of intervention	Medication time	Duration
Chu (18)	C57BL/6 J mice (Male)	Hyperoxia-induced ALI	6/6	Sodium acetate (200 mM)	Oral administration	Pre-treatment	3 weeks
Cui (19)	SD rats (Male)	Acute Pancreatitis-induced ALI	10/10	Sodium butyrate (5 mg/kg/d)	Intravenous injection	Post-treatment	12 h
Ding (20)	SD rats (Male)	LPS-induced ALI	10/10	Sodium butyrate (1 g/kg/d)	Intravenous injection	Post-treatment	6 h
Hildebrand (2021) (26)	C57BL/6 mice (Male)	LPS-induced ALI	12/12	Sodium acetate, propionate, butyrate (50 mM each)	Oral administration	Pre-treatment	2 weeks
Hung (27)	SD rats (Male)	I/R-Induced ALI	6/6	Sodium acetate (100 mg/kg/d, 200 mg/kg/d, 400 mg/kg/d)	Pulmonary artery perfusion	Post-treatment	60 min
Li (28)	BALB/c mice (Male)	LPS-induced ALI	10/10	Sodium butyrate (500 mg/kg)	Intraperitoneal injection	Pre-treatment	24 h
Liang (29)	SD rats (Female)	Burn-induced ALI	8/8	Sodium butyrate (400 mg/kg/d)	Intraperitoneal injection	Post-treatment	12 h, 24 h, 48 h
Liu (31)	ICR mice (Female)	LPS-induced ALI	10/10	Sodium butyrate (25 mg/kg/d)	Intragastric administration	Pre-treatment	12 h
Ni (30)	BALB/c mice (Male)	LPS-induced ALI	3/3	Sodium butyrate (10 mg/kg/d)	Intragastric administration	Pre-treatment	1 h, 3 h, 6 h, 12 h, 24 h
Tang (32)	SD rats (Male)	Intestinal I/R-induced ALI	10/10	Sodium butyrate (400 mg/kg/d)	Subcutaneous injection	Post-treatment	1 h, 4 h
Xiang (2022) (33)	SD rats (Male)	LPS-induced ALI	5/5	Sodium acetate, propionate, butyrate (300 mg/kg/d, 100 mg/kg/d, 100 mg/kg/d; 600 mg/kg/d, 200 mg/kg/d, 200 mg/kg/d)	Intragastric administration	Pre-treatment	7 days
Xiong (34)	BALB/c mice (Male)	Acute Pancreatitis-induced ALI	8/8	Sodium butyrate (200 mg/kg/d, 500 mg/kg/d)	Intragastric administration	Pre-treatment	7 days
Xu (35)	C57BL/6 J mice (Male)	LPS-induced ALI	10/10	Sodium acetate (4 mmol/kg/d)	Intraperitoneal injection	Post-treatment	6 h
Yang (36)	C57BL/6 J mice (Male)	CLP-induced ALI	6/6	Sodium butyrate (25 mg/kg/d)	Intragastric administration	Pre-treatment, Post-treatment	24 h
Ying (37)	C57BL/6 J mice (Male)	I/R-Induced ALI	NA	Sodium butyrate (5 mg/kg/d)	NA	Pre-treatment	1 week
Zhang L (38)	C57BL/6 J mice (Male)	CLP-induced ALI	6/6	Sodium butyrate (200 mg/kg/d)	Intraperitoneal injection	Pre-treatment	18 h
Zhang TT (21)	SD rats (Male)	LPS-induced ALI	5/5	sodium propionate (300 mg/kg/d, 500 mg/kg/d)	Intragastric administration	Pre-treatment	7 days
Zhang YD (39)	C57BL/6 mice (Male)	ZnONPs-induced ALI	5/5	Sodium acetate (100 mM), Sodium propionate (100 mM)	Oral administration	Pre-treatment	21 days

EG, experimental group; CG, control group; LPS, lipopolysaccharides; CLP, cecal ligation and puncture; I/R, ischemia/reperfusion; NA, not applicable.

### 3.2 Risk of bias assessment

**Figure 2:**
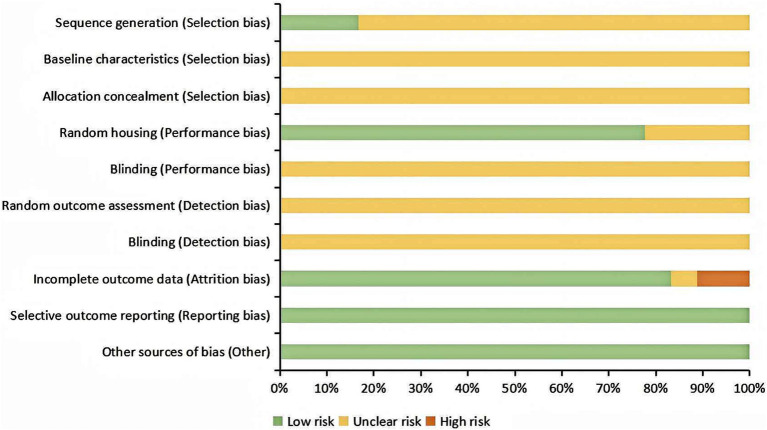
Risk of bias assessment results.

The overall result is shown in Figure 2 and Supplementary Table S3. Of the 18 articles included, only three clearly reported the use of a random number table for animal randomization (20, 33, 36), while the remaining articles merely mentioned “randomization” without specifying the method and were therefore rated as “unclear.” Based on the descriptions of animal housing conditions in 14 articles (18–21, 26, 28–30, 33, 35–39), it could be concluded that animals were randomly housed during the experiments. In the integrity report entries, we found that two articles did not provide accurate sample size data, thereby potentially posing a higher risk of bias (28, 37). To ensure the reliability of the analysis results, we only recorded the characteristics of these two studies and did not include their experimental data. In the end, a total of 16 articles were included in this meta-analysis.

### 3.3 Meta-analysis of primary outcomes

#### 3.3.1 Lung W/D ratio

**Figure 3:**
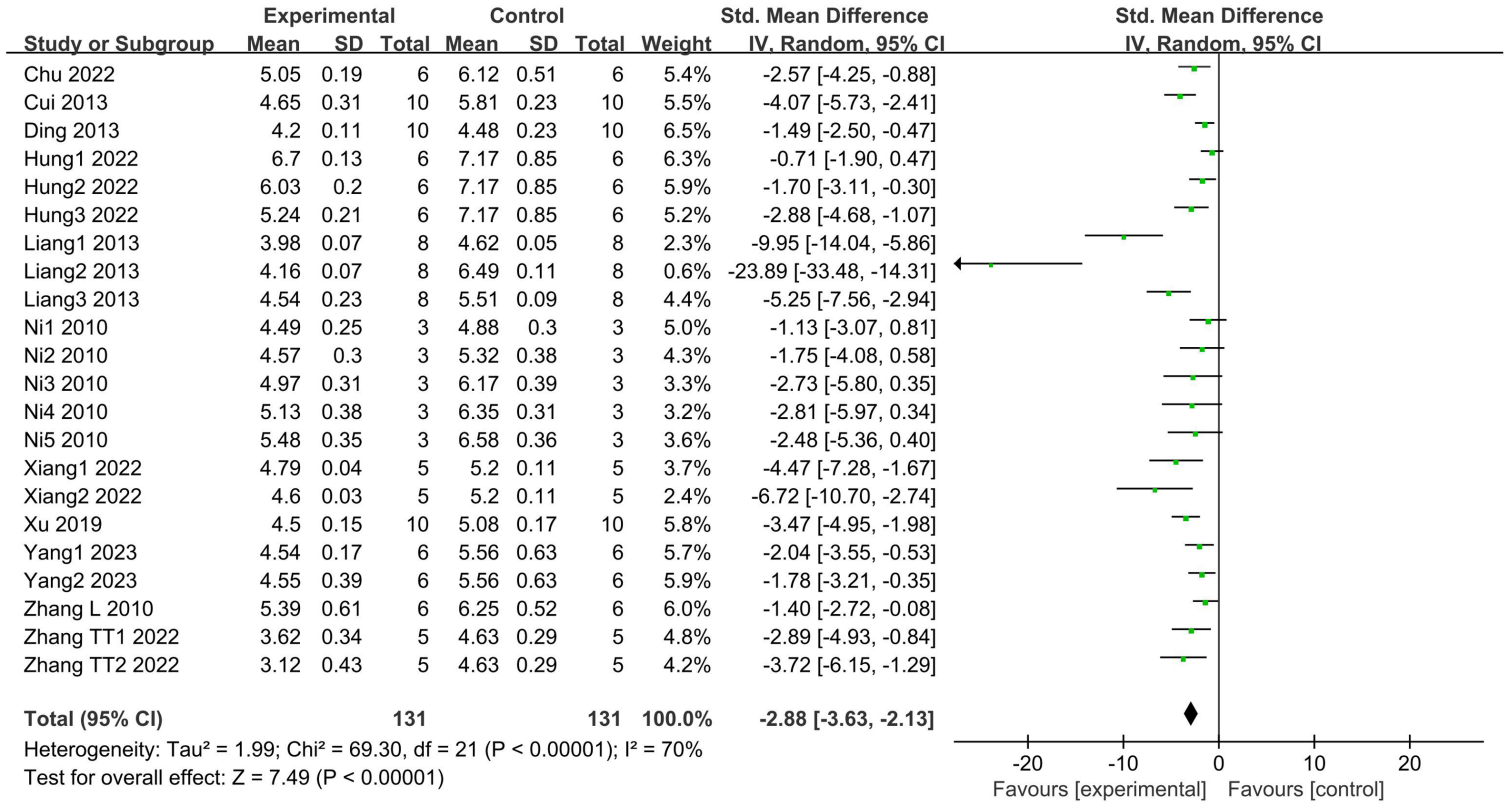
Forest plot of the effects of SCFAs intervention on the lung W/D ratio.

Lung W/D ratio is a direct indicator of pulmonary edema and an important measure for assessing the severity of ALI (40). A total of 11 articles (18–21, 27, 29, 31, 33, 35, 36, 38), including 22 studies, reported the levels of W/D ratios. The results indicated that the SCFAs intervention significantly reduced the lung W/D ratio (I^2^ = 70%; SMD = −2.88, 95% CI = −3.63 to −2.13, *p* < 0.00001) in comparison to the control group (Figure 3).

#### 3.3.2 Lung injury scores

**Figure 4:**
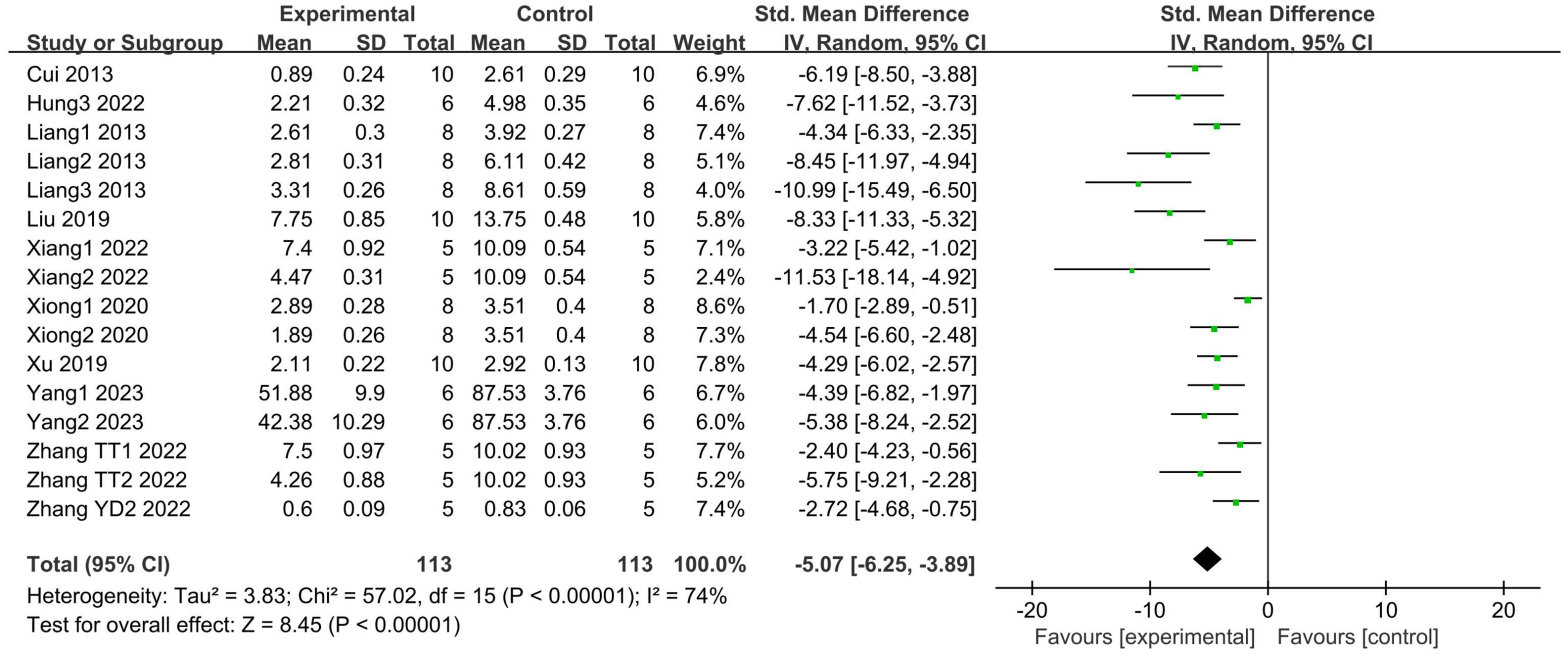
Forest plot of the effects of SCFAs intervention on the lung injury scores.

A total of 10 articles (19, 21, 27, 29, 30, 33–36, 39), which include 16 studies, reported changes in lung injury scores. The results indicate that SCFAs intervention can effectively reduce lung injury scores (I^2^ = 74%; SMD = −5.07, 95% CI = −6.25 to −3.89, *p* < 0.00001) compared to the control group (Figure 4).

### 3.4 Meta-analysis of secondary outcomes

#### 3.4.1 MPO

**Figure 5:**
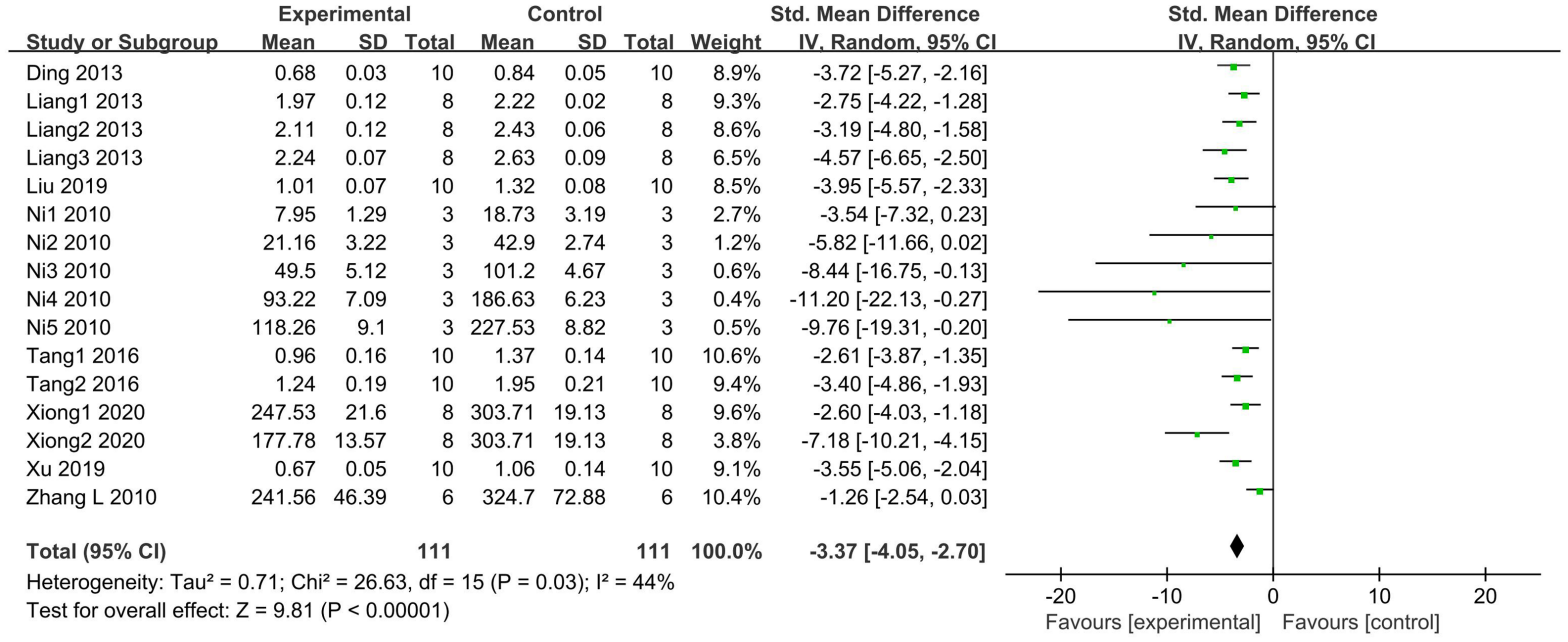
Forest plot of the effects of SCFAs intervention on MPO activity in lung tissue.

MPO serves as a marker of neutrophil activation and is significantly increased in tissue injury and various associated inflammatory diseases (41). A total of 8 articles (20, 29–32, 34, 35, 38), including 16 studies, compared lung tissue MPO activities between the SCFAs and control groups. The SCFAs intervention groups showed lower MPO activities (I^2^ = 44%; SMD = −3.37, 95% CI = −4.05 to −2.70, *p* < 0.00001) than the control group (Figure 5).

#### 3.4.2 TNF-*α*

**Figure 6:**
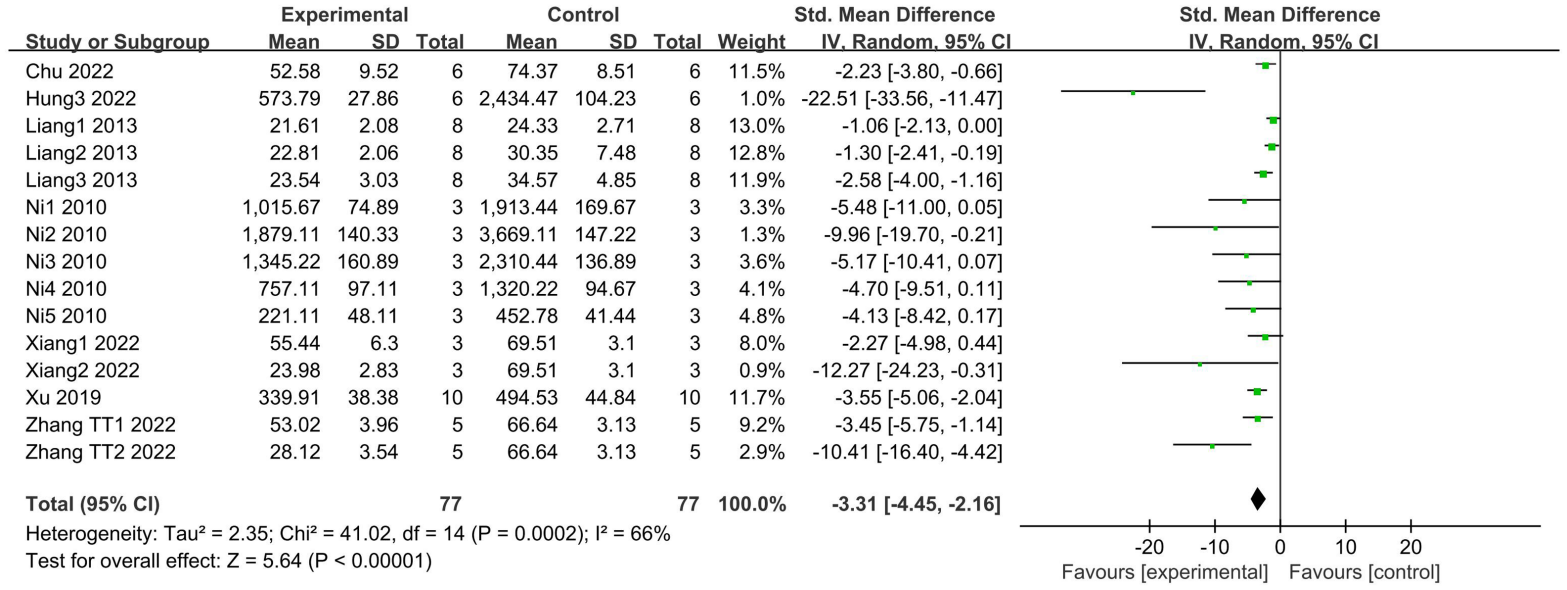
Forest plot of the effects of SCFAs intervention on TNF-α in BALF.

TNF-α is a pleiotropic cytokine that leads to the development of inflammatory responses in ALI (42). A total of 7 articles (18, 21, 27, 29, 31, 33, 35), including 15 studies, reported changes in TNF-α levels in the bronchoalveolar lavage fluid (BALF). Compared to the control group, the SCFAs intervention could significantly reduce the TNF-α levels (I^2^ = 66%; SMD = −3.31, 95% CI = −4.45 to −2.16, *p* < 0.00001; Figure 6).

#### 3.4.3 MDA

**Figure 7:**
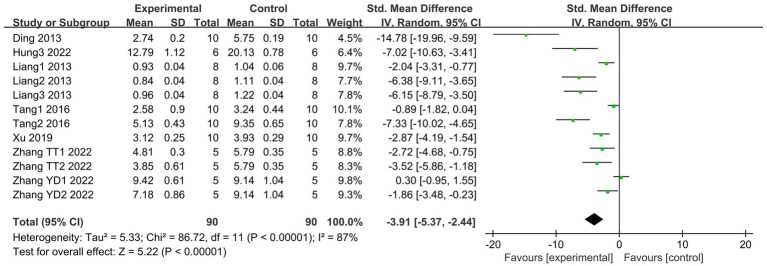
Forest plot of the effects of SCFAs intervention on MDA level in lung tissue.

MDA is one of the most frequently examined biomarkers of oxidative stress (43). A total of 7 articles (20, 21, 27, 29, 32, 35, 39), including 12 studies, compared lung tissue MDA levels between SCFAs and control groups. The SCFAs intervention significantly alleviated MDA expression (I^2^ = 87%; SMD = −3.91, 95% CI = −5.37 to −2.44, *p* < 0.00001) compared to the control group (Figure 7).

### 3.5 Subgroup analysis of primary outcome indicators

We conducted subgroup analyses basing on the following variables: the modeling methods, types of SCFAs, dosage, duration, animal species, and administration route. For the dosage subgroup analysis, we excluded one study because it did not define the drug dosage (39).

#### 3.5.1 Lung W/D ratio

The results of the subgroup analysis showed that the 400 mg/kg/d dosage group may have a more significant effect in reducing W/D levels (I^2^ = 88%; SMD = −8.31, 95% CI = −13.02 to −3.59, *p* = 0.0006) compared to other dosages. Additionally, intervention duration exceeding 24 h may provide better results (I^2^ = 24%; SMD = −3.81, 95% CI = −4.91 to −2.70, *p* < 0.00001) compared to shorter treatment durations. The analysis also indicated that SCFAs showed better efficacy in rat models (I^2^ = 82%; SMD = −3.90, 95% CI = −5.28 to −2.52, *p* < 0.00001). There were no statistically significant differences between the subgroups concerning the modeling method, type of SCFAs, and administration route (*p* > 0.05). The results of all subgroups were consistent with the overall results (Table 2; Supplementary Figures S1–S6).

**Table 2:** Subgroup analysis for lung W/D ratio.

Variables	Studies	SMD (95% CI)	*I^2^*	*p* for overall effect	*p* for subgroup differences
Total	22	−2.88 (−3.63, −2.13)	70%	<0.00001	
Modeling method					
LPS-induced ALI	11	−2.67 (−3.48, −1.86)	34%	<0.00001	0.04
CLP-induced ALI	3	−1.71 (−2.53, −0.90)	0%	<0.0001	
Others	8	−4.12 (−6.01, −2.24)	86%	<0.0001	
Type of SCFAs					
Sodium acetate	5	−2.18 (−3.22, −1.14)	59%	<0.0001	0.11
Sodium propionate	2	−3.23 (−4.80, −1.67)	0%	<0.0001	
Sodium butyrate	13	−3.01 (−4.17, −1.86)	76%	<0.00001	
Mixed SCFAs	2	−5.22 (−7.52, −2.93)	0%	<0.00001	
Dosage					
≤100 mg/kg/d	9	−1.99 (−2.76, −1.22)	33%	<0.00001	0.05
200-350 mg/kg/d	5	−2.51 (−3.54, −1.48)	46%	<0.00001	
400 mg/kg/d	4	−8.31 (−13.02, −3.59)	88%	0.0006	
≥500 mg/kg/d	3	−3.45 (−6.16, −0.73)	76%	0.01	
Duration					
<12 h	8	−1.84 (−2.54, −1.15)	34%	<0.00001	0.005
12-24 h	8	−3.75 (−5.57, −1.93)	82%	<0.0001	
>24 h	6	−3.81 (−4.91, −2.70)	24%	<0.00001	
Species					
Rats	12	−3.90 (−5.28, −2.52)	82%	<0.00001	0.02
Mice	10	−2.12 (−2.69, −1.55)	0%	<0.00001	
Administration route					
Intragastric administration	11	−2.46 (−3.16, −1.76)	9%	<0.00001	0.08
Intraperitoneal injection	5	−6.23 (−9.52, −2.94)	90%	0.0002	
Intravenous injection	2	−2.69 (−5.22, −0.17)	85%	0.04	
Others	4	−1.81 (−2.80, −0.82)	44%	0.0004	

#### 3.5.2 Lung injury scores

In 15 studies involving drug dosage, we found that a dosage of 400 mg/kg/d was more effective in reducing lung injury scores (I^2^ = 69%; SMD = −7.46, 95% CI = −10.48 to −4.45, *p* < 0.00001) compared to other dosage groups. Additionally, a treatment duration of 24 h to 7 days may yield more pronounced effects (I^2^ = 64%; SMD = −6.87, 95% CI = −9.55 to −4.18, *p* < 0.00001). No statistically significant differences were found among the subgroups regarding the modeling method, type of SCFAs, animal species, and administration route (*p* > 0.05; Table 3; Supplementary Figures S7–S12).

**Table 3:** Subgroup analysis for lung injury scores.

Variables	Studies	SMD (95% CI)	*I^2^*	*p* for overall effect	*p* for subgroup differences
Total	16	−5.07 (−5.25, −3.89)	74%	<0.00001	
Modeling method					
LPS-induced ALI	6	−5.03 (−7.01, −3.05)	71%	<0.00001	0.93
CLP-induced ALI	2	−4.81 (−6.66, −2.96)	0%	<0.00001	
Others	8	−5.30 (−7.21, −3.40)	82%	<0.00001	
Type of SCFAs					
Sodium acetate	2	−5.48 (−8.61, −2.35)	58%	0.0006	0.15
Sodium propionate	3	−3.10 (−4.67, −1.53)	31%	0.0001	
Sodium butyrate	9	−5.65 (−7.45, −3.84)	81%	<0.00001	
Mixed SCFAs	2	−6.77 (−14.82, 1.29)	82%	0.1	
Dosage					
≤100 mg/kg/d	4	−5.94 (−7.49, −4.40)	28%	<0.00001	0.001
200-350 mg/kg/d	4	−2.79 (−4.01, −1.57)	52%	<0.00001	
400 mg/kg/d	4	−7.46 (−10.48, −4.45)	69%	<0.00001	
≥500 mg/kg/d	3	−6.01 (−8.93, −3.09)	5%	<0.0001	
Duration					
≤12 h	5	−5.72 (−7.25, −4.20)	50%	<0.00001	0.02
24-48 h	4	−6.87 (−9.55, −4.18)	64%	<0.00001	
≥7 days	7	−3.42 (−4.77, −2.07)	62%	<0.0001	
Species					
Rats	9	−5.97 (−7.78, −4.16)	71%	<0.00001	0.14
Mice	7	−4.22 (−5.73, −3.89)	84%	<0.00001	
Administration route					
Intragastric administration	9	−4.51 (−6.06, −2.96)	74%	<0.00001	0.48
Intraperitoneal injection	4	−6.36 (−8.94, −3.79)	74%	<0.00001	
Others	3	−5.07 (−6.25, −2,29)	74%	0.0005	

### 3.6 Sensitivity analysis

In order to explore the stability of the results, a sensitivity analysis was conducted on five meta-analyses. Eliminating any single study did not reverse the overall effects of SCFAs on the ALI (Supplementary Figure S13). It suggested that the meta-analyses results were relatively stable.

### 3.7 Publication bias

The funnel plots of the five meta-analyses exhibited asymmetry (Supplementary Figure S14), and the results of Egger’s test were statistically significant (*p* < 0.05). While these findings suggested a certain degree of publication bias, the trim-and-fill analyses indicated that publication bias did not affect the overall estimate (Supplementary Figure S15; no trimming was performed and the data remained unchanged). Consequently, the conclusions remained robust.

## 4 Discussion

As the role of gut microbiota and their metabolites in regulating immunity and inflammatory responses becoming increasingly clear, their relationship with lung disease (also known as gut-lung axis) have attracted significant attention (10). SCFAs, produced by gut microbiota fermentation, are regarded as key molecules in the gut-lung axis, providing a novel therapy to alleviate even reverse the ALI deterioration (44, 45). To the best of our knowledge, this is the first systematic review and meta-analysis exploring the effects of SCFAs supplementation on ALI in animal research. The subgroup analysis results indicated that the therapeutic effects of SCFAs may be closely related to the dosage and duration of treatment, while being less influenced by the modeling method and the type of SCFAs. In particular, when the dosage reached 400 mg/kg/d and was administered continuously for 24 h to 7 days, the SCFAs showed a better efficacy. Despite the high heterogeneity and publication bias, SCFAs still significantly improved the inflammation levels and lung injury severity in ALI animals based on the overall results. Additionally, we obtained similar results through sensitivity analysis and trim-and-fill analysis, which enhanced the robustness of our findings.

The homeostasis of the gut actually impacts lung health (45). How is this effect mediated by SCFAs? Some studies have explored this in depth.

In fact, there is an immune mechanism regulated by the microbiota that exists between the respiratory and gastrointestinal tracts. The term “gut microbiota” refers to the complex ecological community composed of symbiotic and pathogenic microorganisms residing in the gut, encompassing thousands of species of bacteria, archaea, viruses, fungi, and other microbes (46, 47). These microorganisms maintain a dynamic balance and protect the gut through multifaceted mechanisms, such as synthesizing metabolites and toxins, providing nutrients, regulating the immune system, and preserving the function of the intestinal barrier (48, 49). Once the balance is disrupted, abnormal microorganisms may breach the intestinal barrier, enter the bloodstream, or migrate to the lungs, leading to systemic infections (sepsis) and lung damage (50). Among the various factors influencing gut microbiota, diet is considered one of the most effective interventions (51). Research shows that a high-fiber diet promotes the production of abeneficial metabolites, especially SCFAs (52). SCFAs construct and sustain the host’s intestinal defense system through various mechanisms, including stimulating the expression of intestinal mucins, enhancing the function of tight junction proteins (TJPs), and regulating the survival of intestinal neurons (53–55). In addition, SCFAs can promote the development of B cells and the differentiation and expansion of regulatory T cells (Tregs) (56–58). Subsequently, these lymphocytes migrate from the gut mucosa through the bloodstream and lymphatic system to reach distant effector sites, ultimately eliciting similar immune responses in mucosal areas throughout the body, including the respiratory mucosa (49, 59, 60).

**Figure 8:**
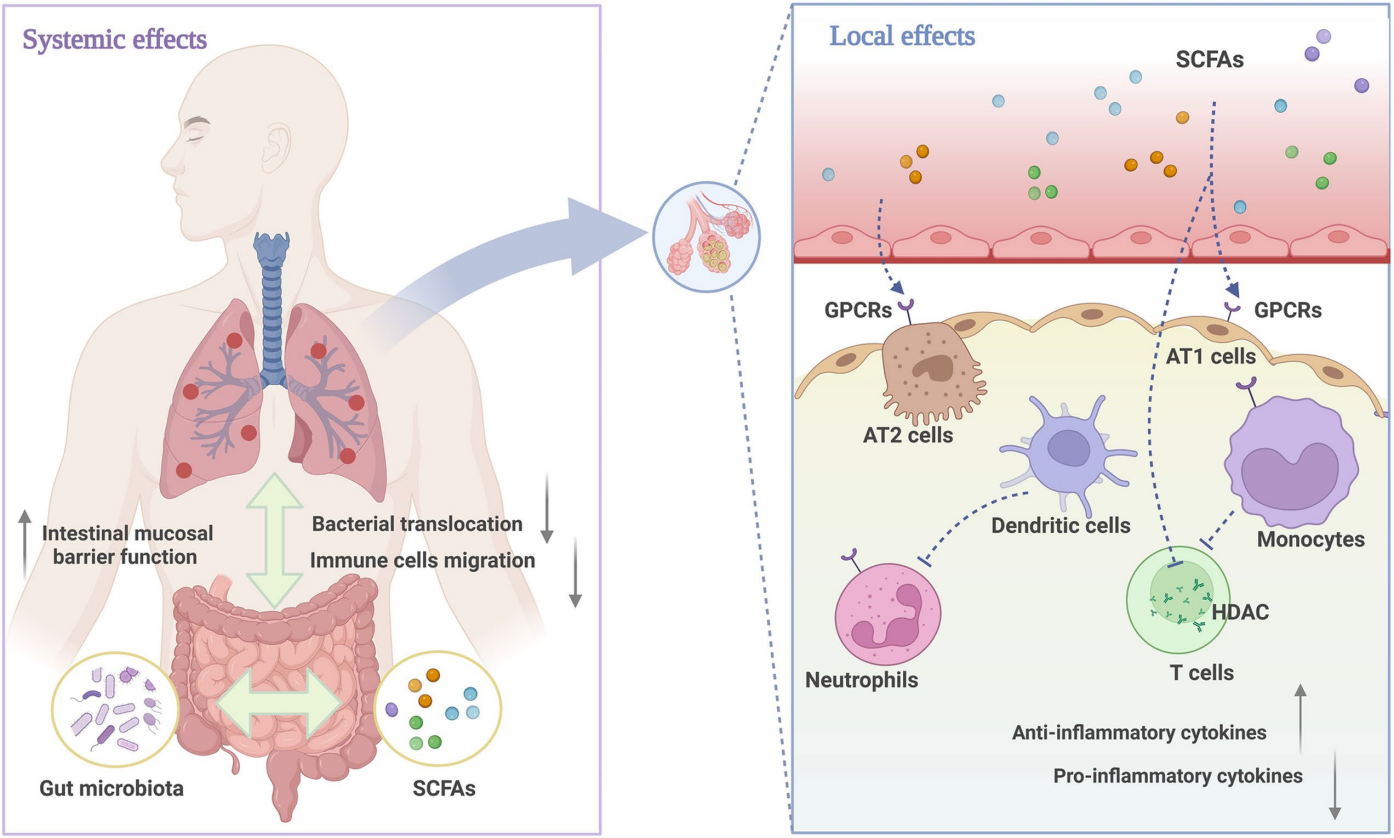
The role of SCFAs in systemic and pulmonary inflammation. The immune mechanisms connecting the respiratory and gastrointestinal tracts are regulated by microbiota, with beneficial metabolites like SCFAs enhancing immune defenses in both systems. Locally, SCFAs act as anti-inflammatory mediators that effectively reduce inflammatory damage. The specific mechanisms include activating GPCRs on alveolar cells, neutrophils, and monocytes, inhibiting HDAC activity to regulate the function of Treg cells, and ultimately affecting the production and release of regulatory cytokines. Created with BioRender.com.

Exceeding 90% of SCFAs are absorbed in the intestines, with a fraction dedicated to sustaining the metabolic functions of intestinal epithelial cells, while the remainder is disseminated through the circulatory system to a variety of organs (61). It has been demonstrated that SCFAs are present in the human respiratory tract, with their levels being closely correlated with the functional integrity of the gut microbiota (44). The capacity of SCFAs to regulate local immune responses is primarily dependent on two mechanisms: firstly, by directly inhibiting histone deacetylases (HDACs) to modulate gene expression, and secondly, by activating G protein-coupled receptors (GPCRs) to transmit signals. The GPCRs that can be activated by SCFAs are GPR41 (also known as FFAR3), GPR43 (also known as FFAR2), and GPR109A (62). These three receptors are widely distributed in various respiratory epithelial cells and immune cells, where they are capable of inhibiting LPS-induced pulmonary inflammatory signaling (63). As a result, they reduce alveolar edema and decrease the production of TNF-*α* in the ALI model (58, 64). However, different SCFA receptors exhibit varying affinities for different SCFAs. GPR41 displays a greater affinity for butyrate and propionate than for acetate, whereas GPR43 exhibits a higher preference for acetate over butyrate and propionate. GPR109A, on the other hand, is primarily activated by butyrate (44). Furthermore, propionate and butyrate are widely recognized as HDAC inhibitors. HDACs are enzymes that remove acetyl groups from histones, leading to the formation of compact chromatin structures that inhibit transcription (65). Thus, the inhibition of HDAC activity can promote gene transcription by increasing histone acetylation. In a mouse model of lung injury induced by polycyclic aromatic hydrocarbons, the restoration of butyrate levels in the gut has been demonstrated to reduce the HDAC level in the lung tissues, enhancing the expression of the Foxp3 gene and regulating Th17/Treg cell differentiation (66). In addition, other studies have shown that butyrate and propionate may reverse lung inflammation and oxidative stress by inhibiting HDAC activity, blocking the nuclear factor (NF)-kappaB signaling pathway, and suppressing the release of MDA in lung tissue (26, 31, 67). A summary of the role of SCFAs in regulating systemic and pulmonary inflammation is provided in Figure 8.

Despite variations in receptor selectivity among different SCFAs that could influence their mechanisms, our findings showed that there are no significant differences in their effectiveness in alleviating ALI. We hypothesized that short-chain fatty acids (SCFAs) may provide a lung-protective effect by activating and integrating various biological signaling pathways, rather than depending solely on the selective activation of a specific receptor. This multi-pathway, cross-receptor mechanism elucidates the synergistic effects of different SCFAs in the body, emphasizing their potential as novel therapeutic strategies for ALI. Nevertheless, further in-depth research is still needed to fully elucidate the mechanisms of action of SCFAs and establish clear causal relationships.

Subgroup analysis indicated that higher dosages of SCFAs and longer treatment durations appeared to be more effective in alleviating pulmonary edema in ALI animal models. SCFAs, as HDAC inhibitors, can silence the transcription of specific inflammatory genes. Previous study reported that SCFAs can only effectively reduce HDAC activity at higher millimolar concentrations (68). High concentrations of butyrate (0.5 mM) directly inhibited the activation, proliferation, and production of cytokines (IFNγ, IL-17) in CD4 T cells by increasing histone acetylation, while low concentrations (0.065 mM) did not exhibit these effects. In comparison, acetate and propionate required higher concentrations (10 mM and 1 mM, respectively) to achieve similar inhibitory effects (22). Similarly, propionate could reshape the metabolic stress and immune function of alveolar macrophages following LPS exposure, contingent on its concentration and timing of administration (44). These findings were consistent with our previous observations and further supported the hypothesis that SCFAs may have a dose-dependent effect. However, the specific dose–response relationship has not yet been clearly defined, which provides important evidence and direction for future studies on the optimal administration of SCFAs.

From the perspective of microbial ecology, SCFAs are primarily produced by the fermentation of dietary fiber and complex carbohydrates by gut bacteria, with Firmicutes and Bacteroidetes playing particularly important roles in this process (69). The two studies by Hildebrand (26) and Xiong (34) collectively revealed key changes in the gut microbiome during lung injury: significant decreases in Lactobacillus, Bacteroides, and Bifidobacterium, which were positively correlated with SCFAs concentrations, providing potential microbiological markers for ALI. It is noteworthy that the interactions between different microorganisms (including bacteria, archaea, and fungi) and their metabolic products also influence the production and diversity of SCFAs (70). For instance, lactic acid can help create an acidic intestinal environment suitable for butyrate-producing bacteria, while pentanoate in feces may originate from symbiotic relationships between archaea and other intestinal bacteria (71, 72). Moreover, SCFAs themselves can feedback-regulate the microbial community, promoting the growth of beneficial bacteria while inhibiting the virulence of intestinal pathogens (73). The interaction between the gut microbiome and SCFAs is a multilayered and complex system. Its mechanisms extend beyond nutrition and metabolism to include immune regulation, interactions among microbial lineages, and ultimately influence gut barrier function and the homeostasis of the gut-lung axis (74–76). However, there is currently no research that can provide a systematic and comprehensive elucidation of the specific processes and interrelationships of these mechanisms. Therefore, it is still necessary to explore strategies for targeting the regulation of the gut microbiome and SCFAs as potential treatments for ALI.

## 5 Limitations

This study still has some limitations that warrant attention in future research. First, the quality scores of the included articles were relatively low, which may have resulted in selection bias, implementation bias, and measurement bias. Although we have taken measures to control these biases, they may still affect the reliability of the study results to some extent. Secondly, following the subgroup analysis, there was no significant improvement in heterogeneity within each subgroup. This may be related to the limited number of studies and insufficient variable information in the literature, which could have led to the omission of some potential factors. To more accurately determine the optimal treatment strategy, we recommend conducting more high-quality, multidimensional studies in the future to extensively investigate relevant variables and thoroughly analyze the dose-dependent efficacy of SCFAs. Additionally, we expect subsequent research to employ more standardized experimental designs and evaluation indicators to enhance the overall quality of animal experiments, thereby providing reliable scientific evidence for clinical applications.

## 6 Conclusion

This meta-analysis highlights the therapeutic potential of SCFAs in animal models of ALI. The overall evidence supports that SCFAs can alleviate the severity of ALI, suppress inflammatory responses, and reduce oxidative stress levels. Subgroup analyses further suggest that SCFAs may have a dose-dependent effect. These findings will provide important directions for further research and clinical applications in this field.

## Data Availability

The original contributions presented in the study are included in the article/Supplementary material, further inquiries can be directed to the corresponding authors.
