# Supplementary material for: Therapeutic potential of short-chain fatty acids for acute lung injury: a systematic review and meta-analysis of preclinical animal studies
Source: Front Nutr. 2025 Jan 8;11:1528200. doi: 10.3389/fnut.2024.1528200 (PMC11752998; doi:10.3389/fnut.2024.1528200)
Supplement: Supplementary file 1 [file Data_Sheet_1.docx]

Supplementary Material

# Supplementary Figures and Tables

## Supplementary Figures


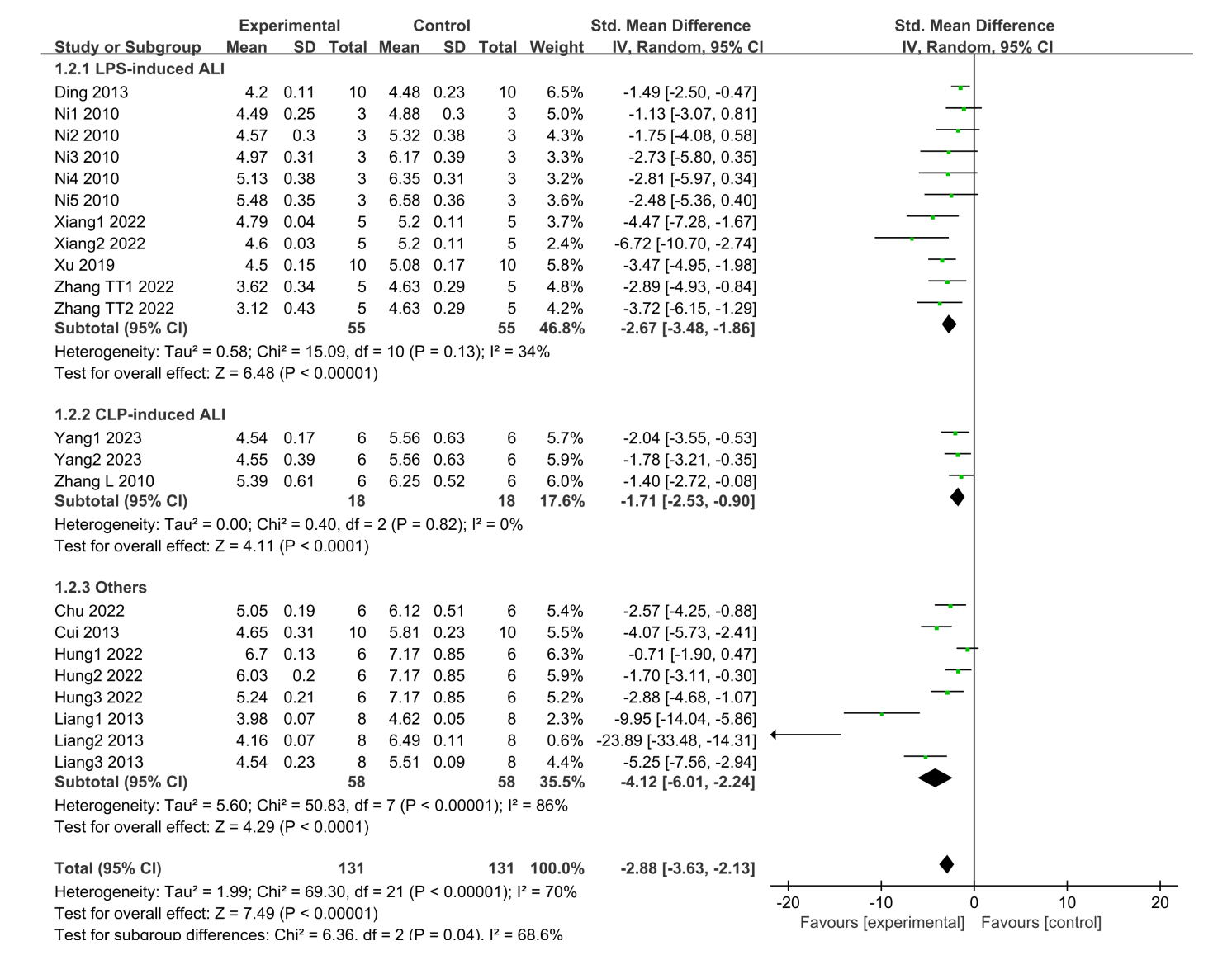


**Supplementary Figure 1.** Forest plot of subgroup analysis of the effect of SCFAs intervention on the lung W/D ratio based on modeling method.


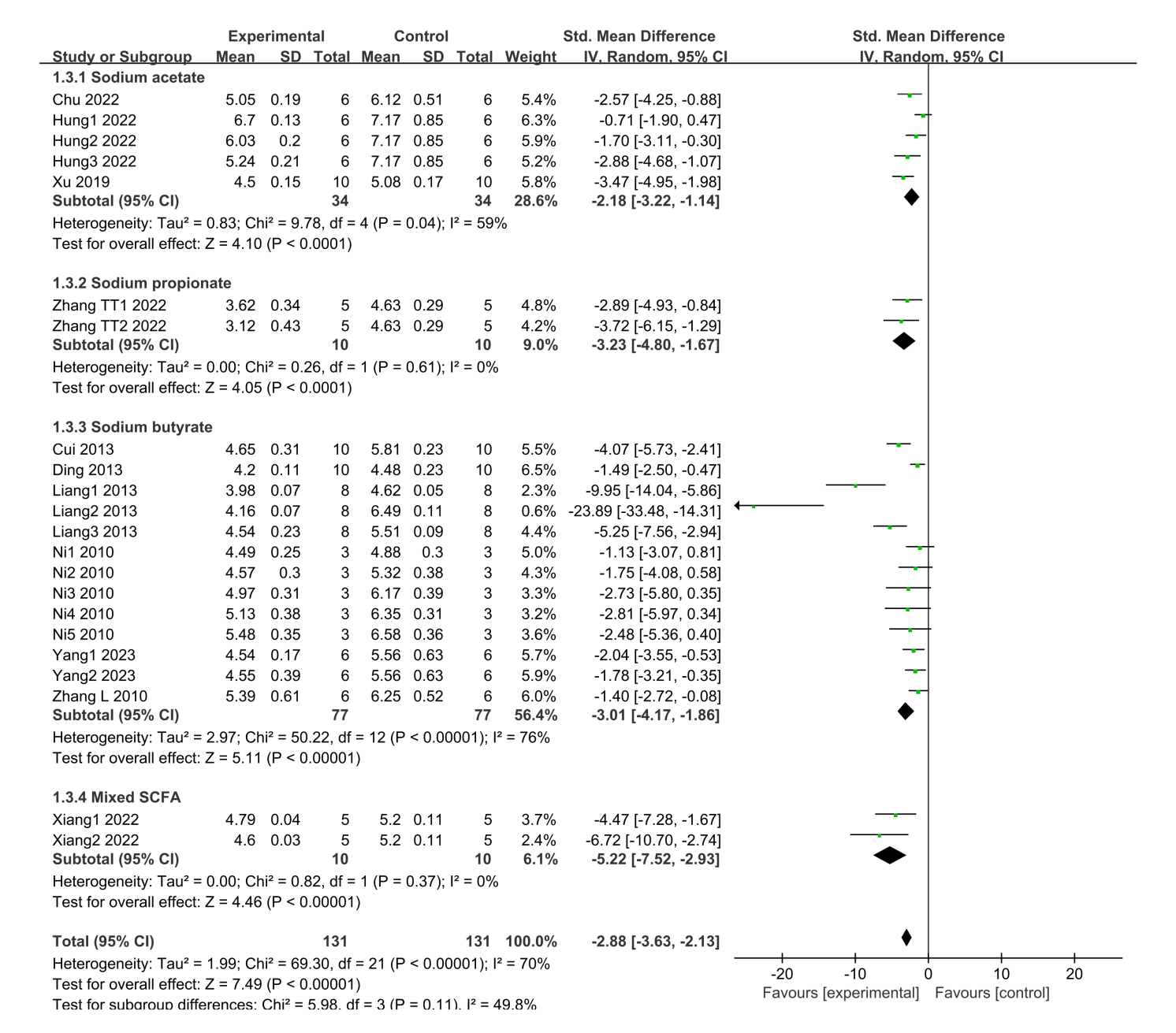


**Supplementary Figure 2.** Forest plot of subgroup analysis of the effect of SCFAs intervention on the lung W/D ratio based on the type of SCFAs.


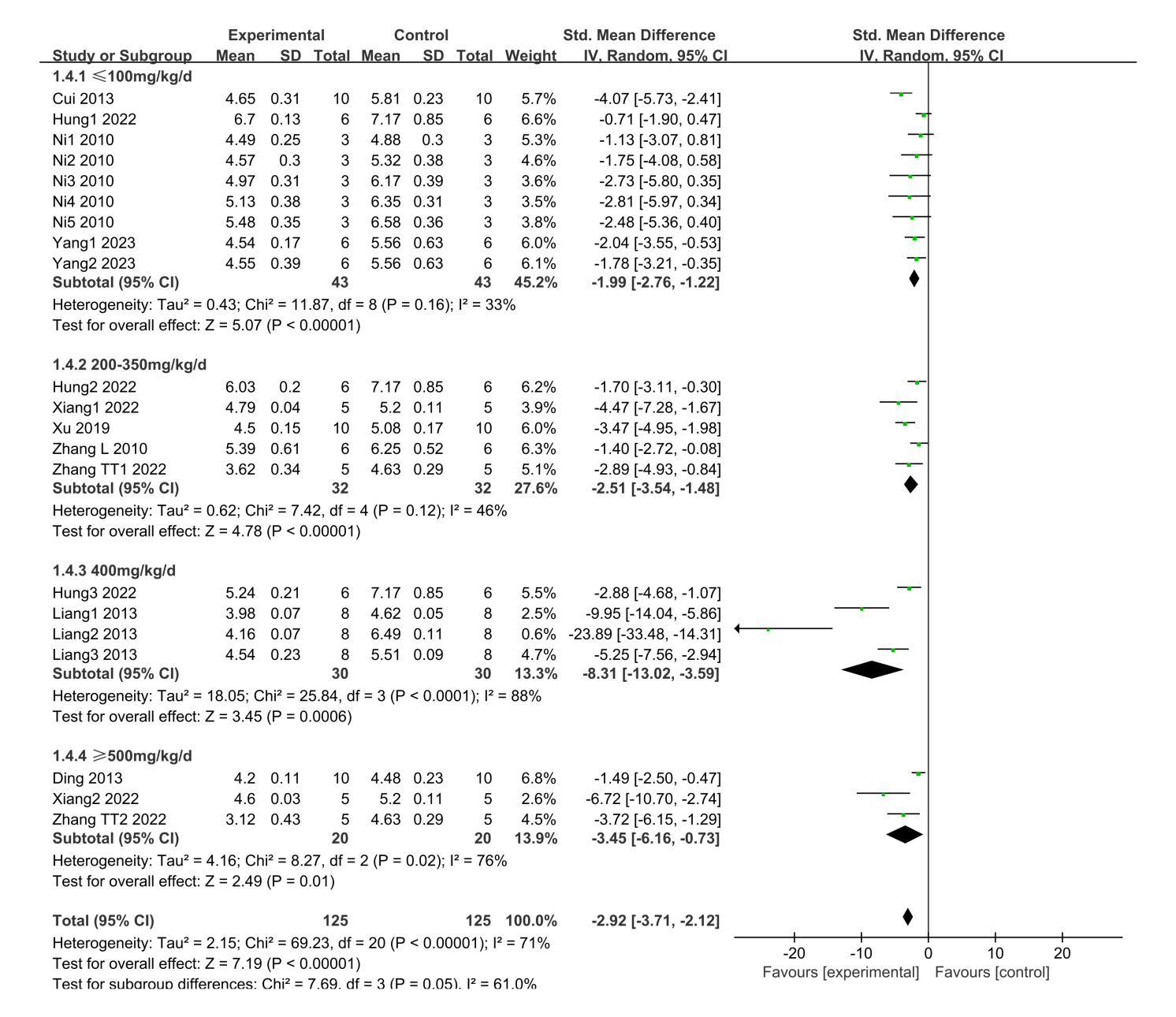
**Supplementary Figure 3.** Forest plot of subgroup analysis of the effect of SCFAs intervention on lung W/D ratio based on dosage.


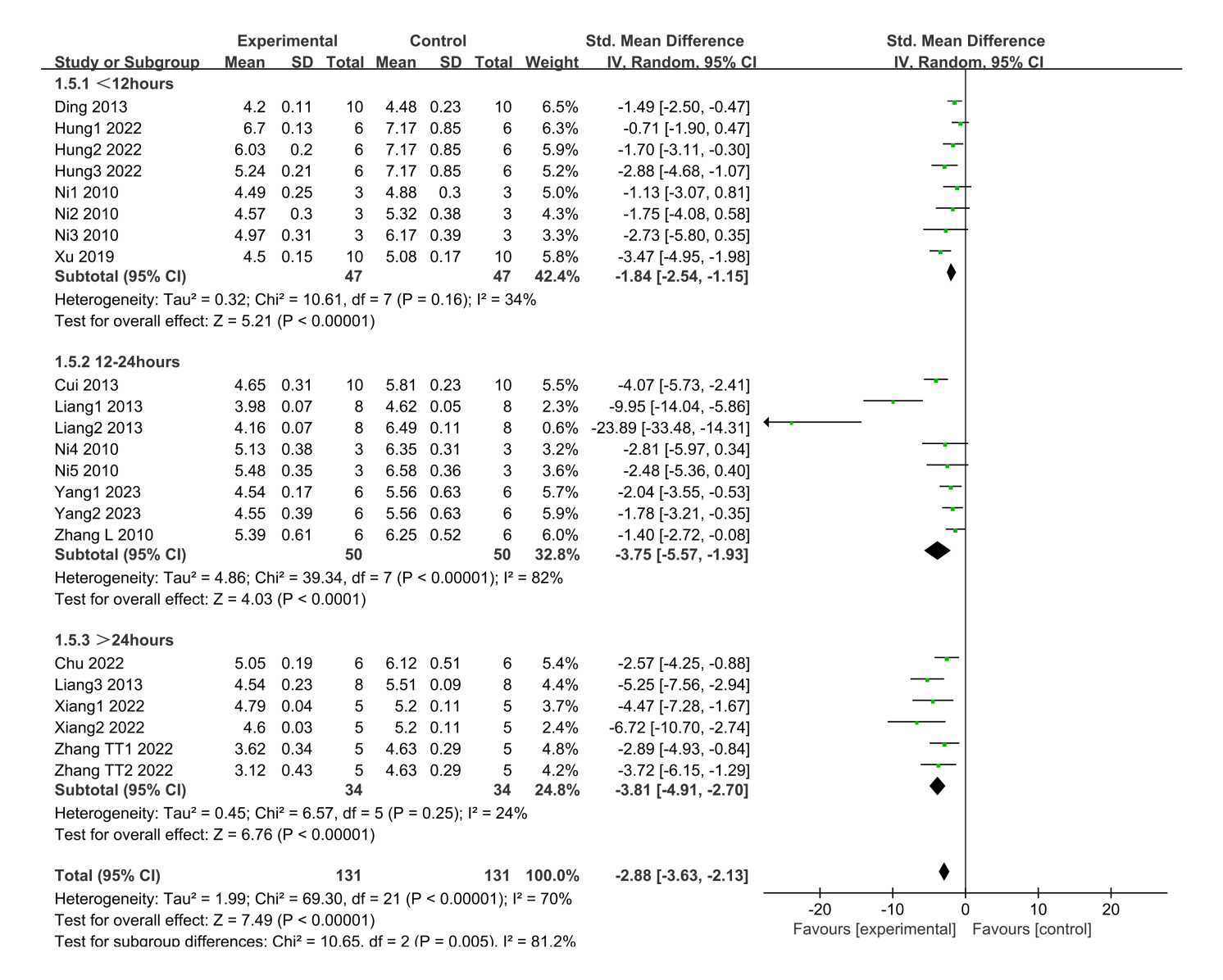
**Supplementary Figure 4.** Forest plot of subgroup analysis of the effect of SCFAs intervention on lung W/D ratio based on duration.


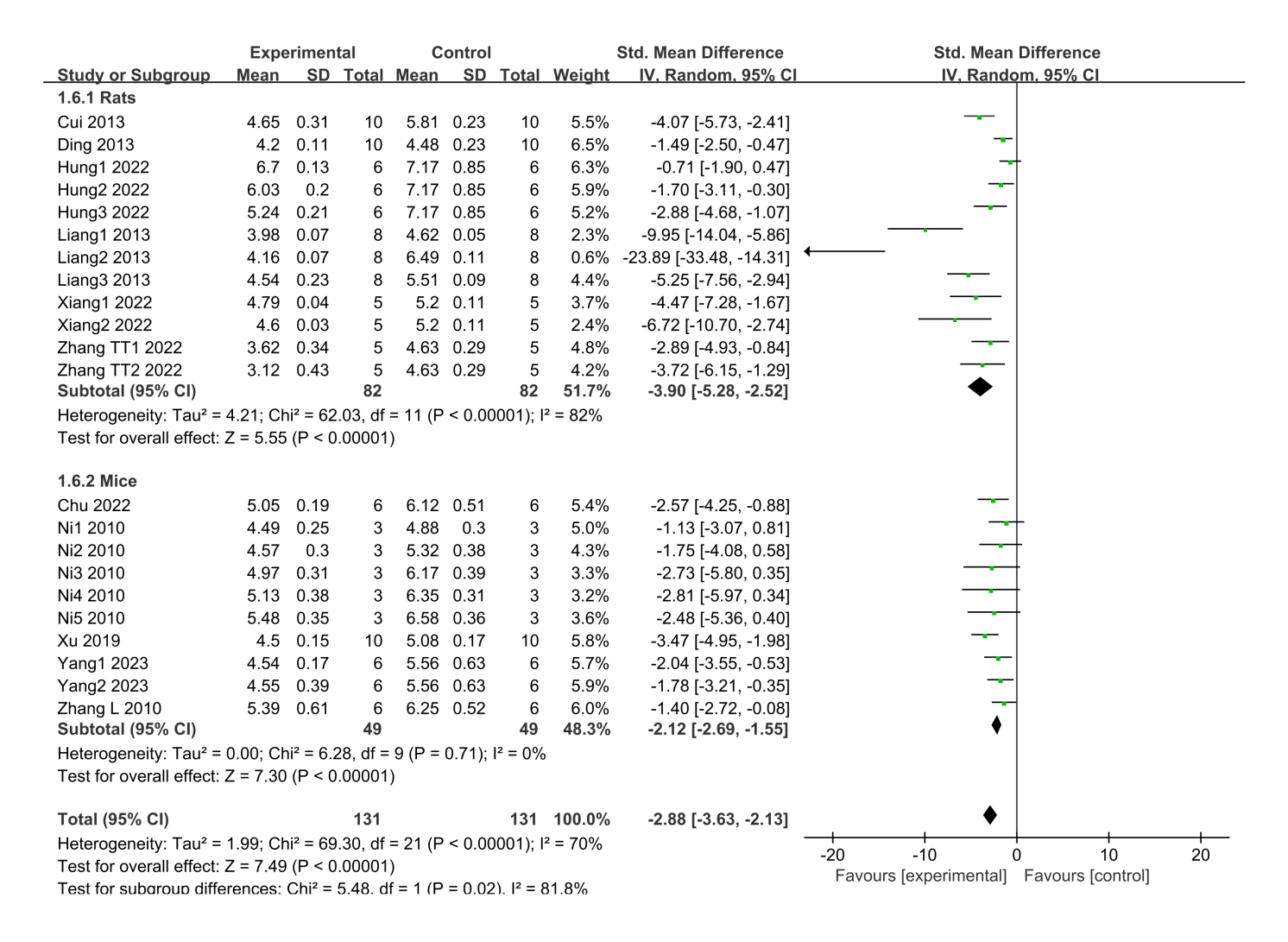
**Supplementary Figure 5.** Forest plot of subgroup analysis of the effect of SCFAs intervention on lung W/D ratio based on animal species.


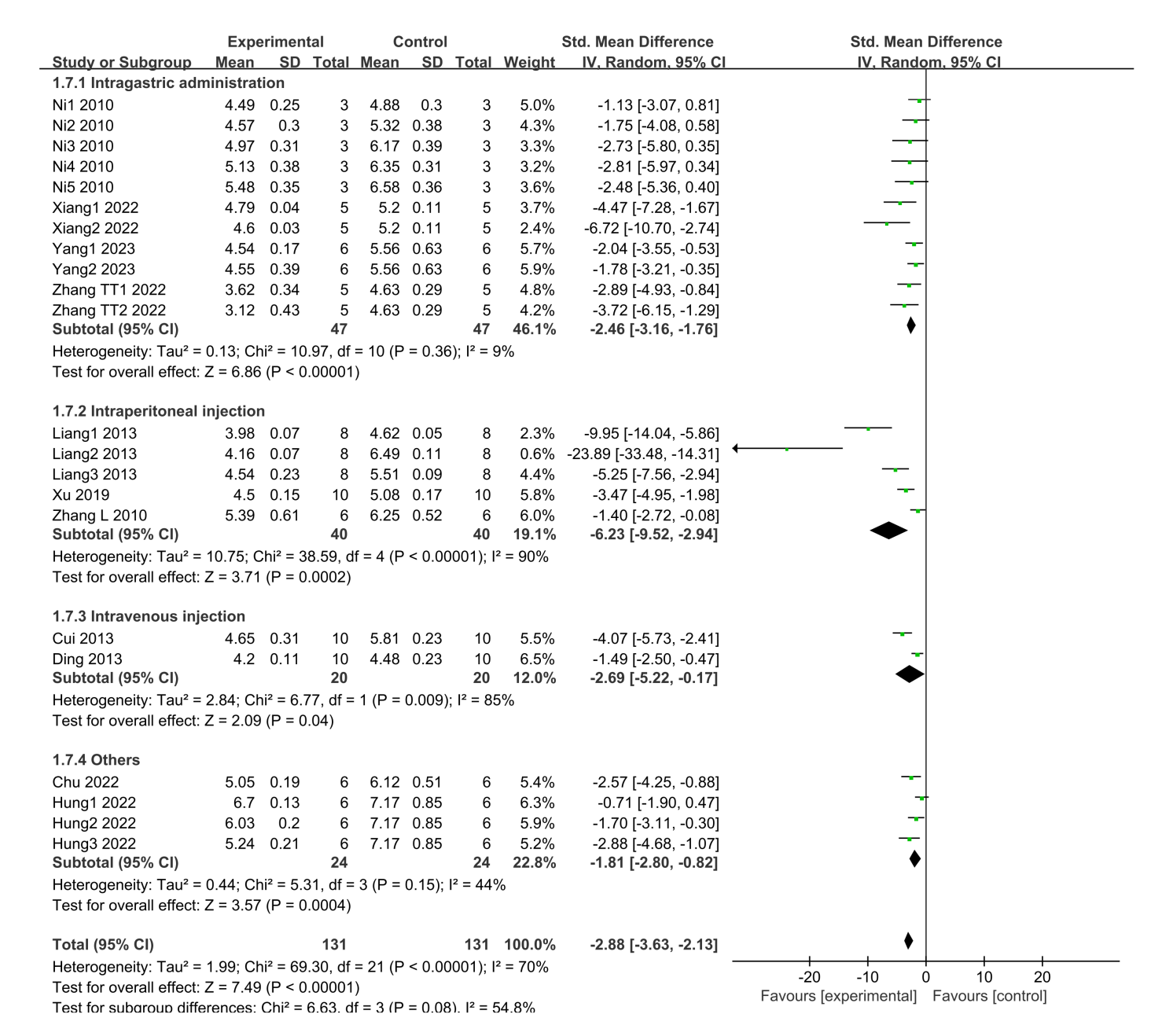
**Supplementary Figure 6.** Forest plot of subgroup analysis of the effect of SCFAs intervention on lung W/D ratio based on administration route.


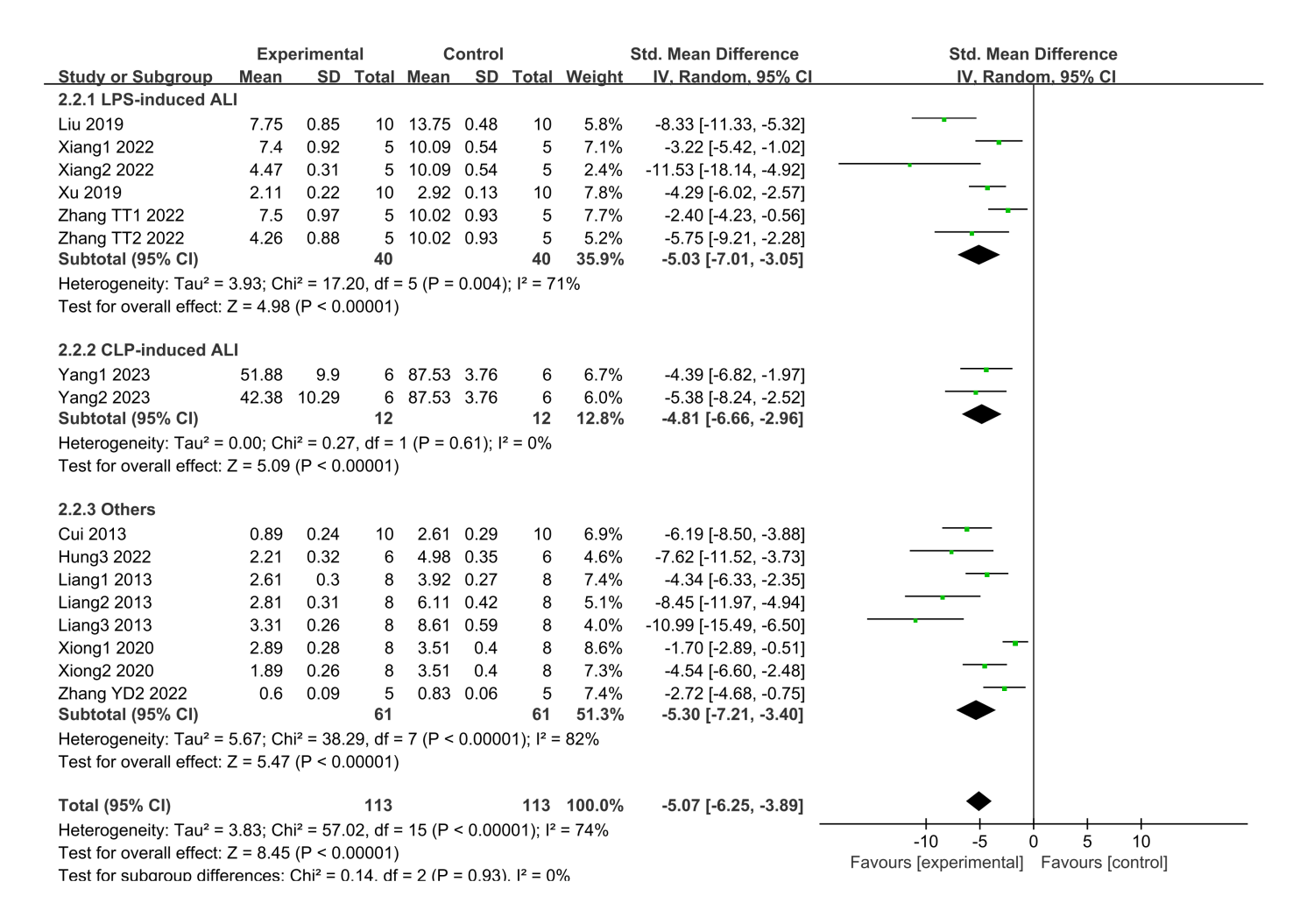
**Supplementary Figure 7.** Forest plot of subgroup analysis of the effect of SCFAs intervention on the lung injury scores based on modeling method.


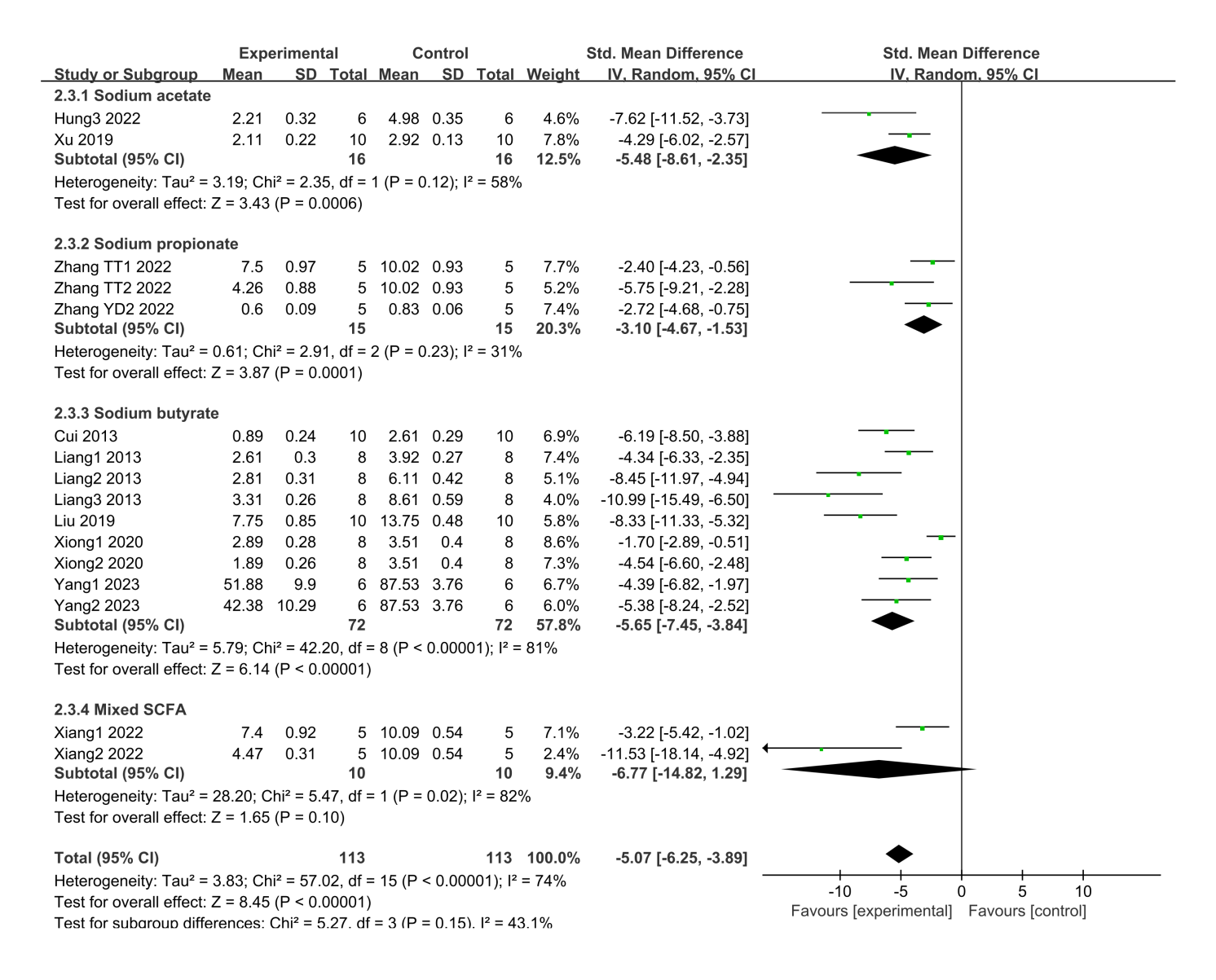
**Supplementary Figure 8.** Forest plot of subgroup analysis of the effect of SCFAs intervention on the lung injury scores based on the type of SCFAs.


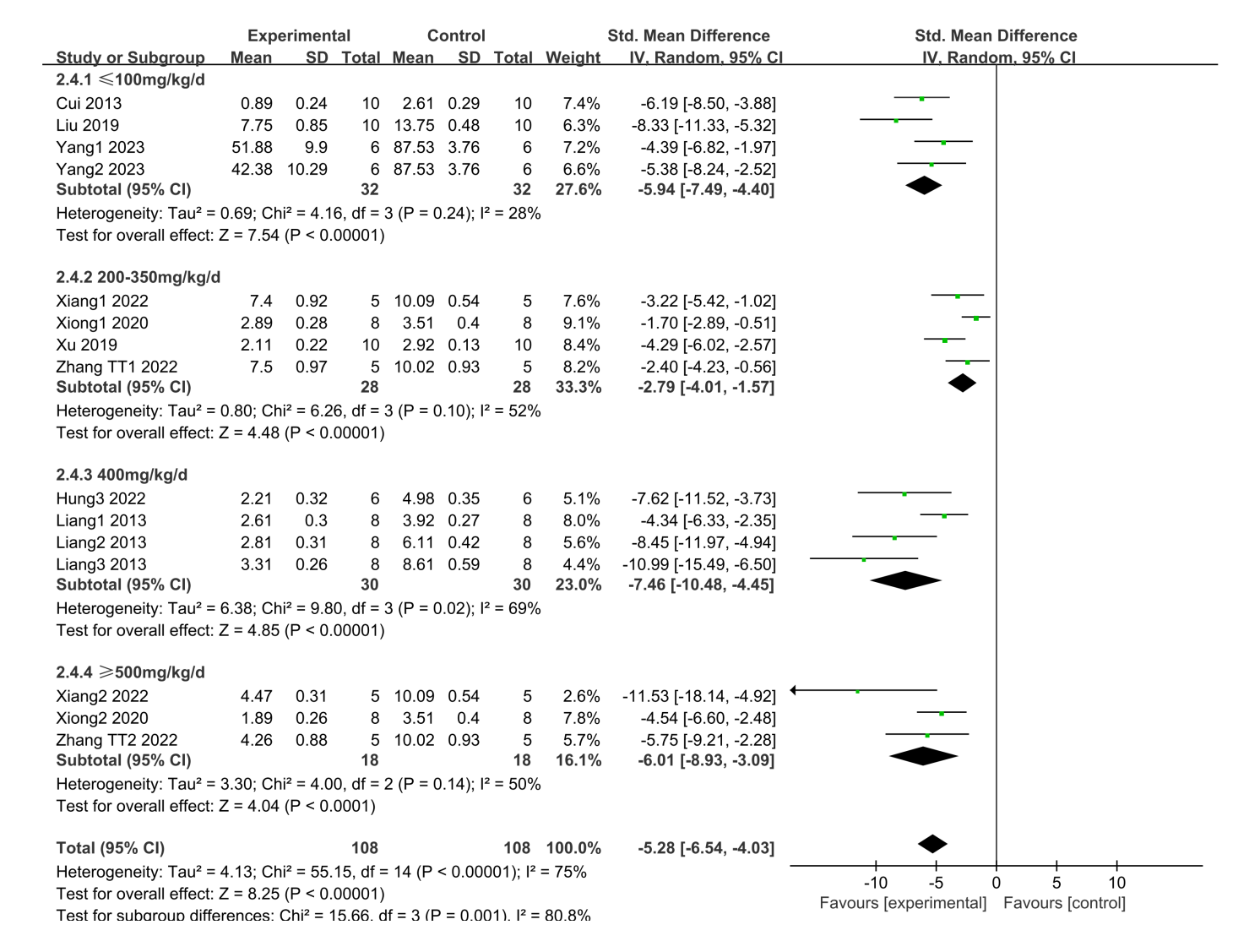
**Supplementary Figure 9.** Forest plot of subgroup analysis of the effect of SCFAs intervention on lung injury scores based on dosage.


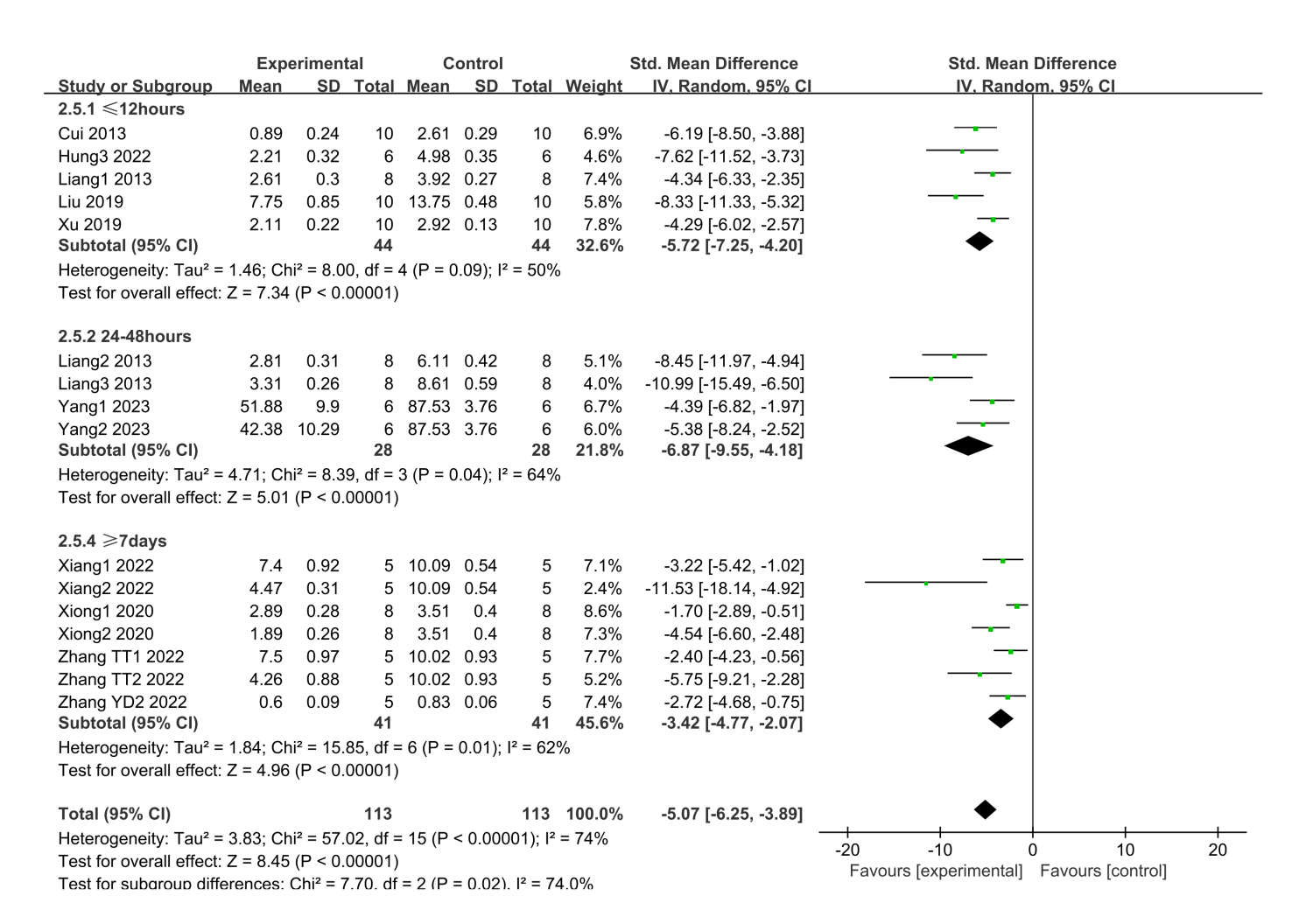
**Supplementary Figure 10.** Forest plot of subgroup analysis of the effect of SCFAs intervention on lung injury scores based on duration.


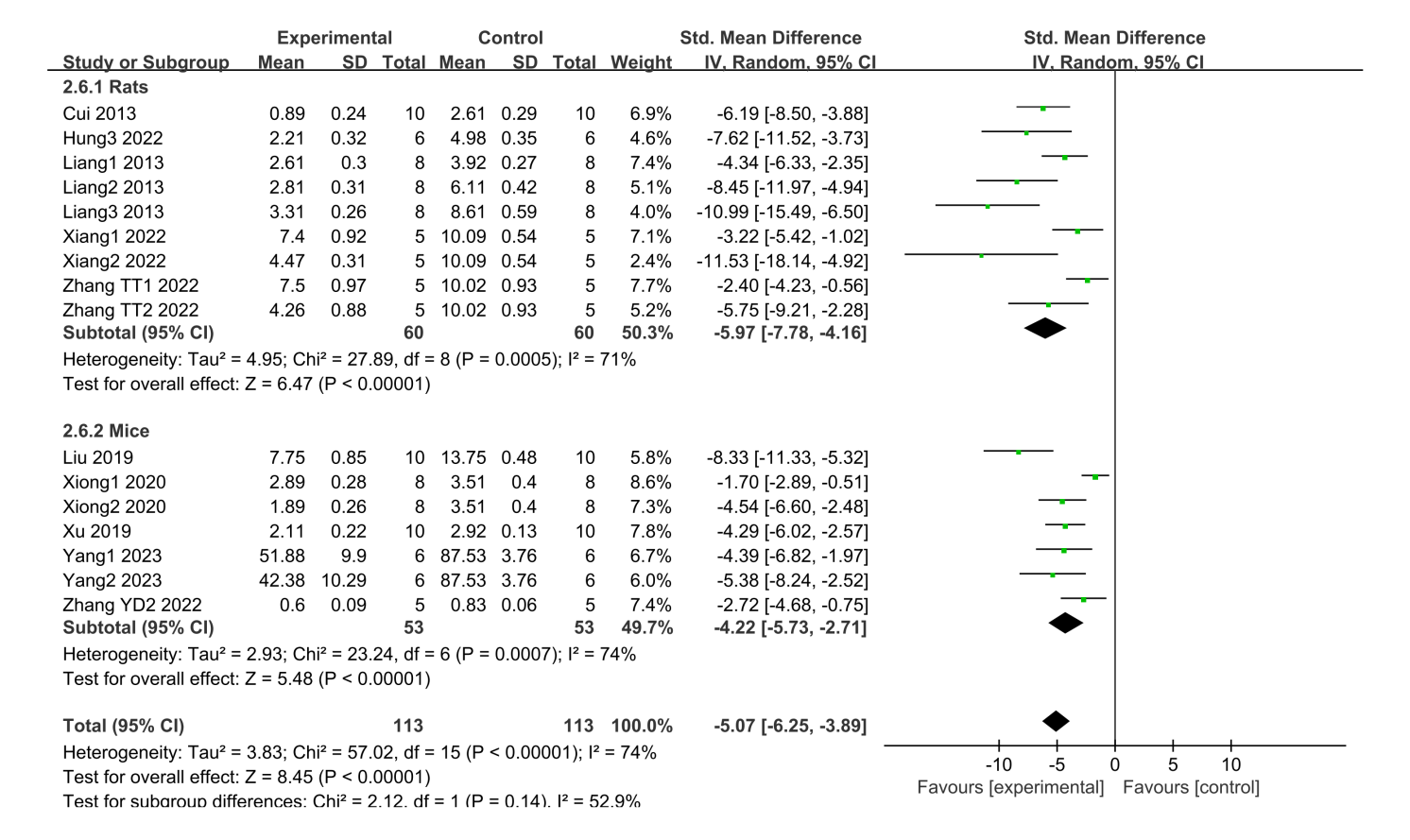
**Supplementary Figure 11.** Forest plot of subgroup analysis of the effect of SCFAs intervention on lung injury scores based on animal species.


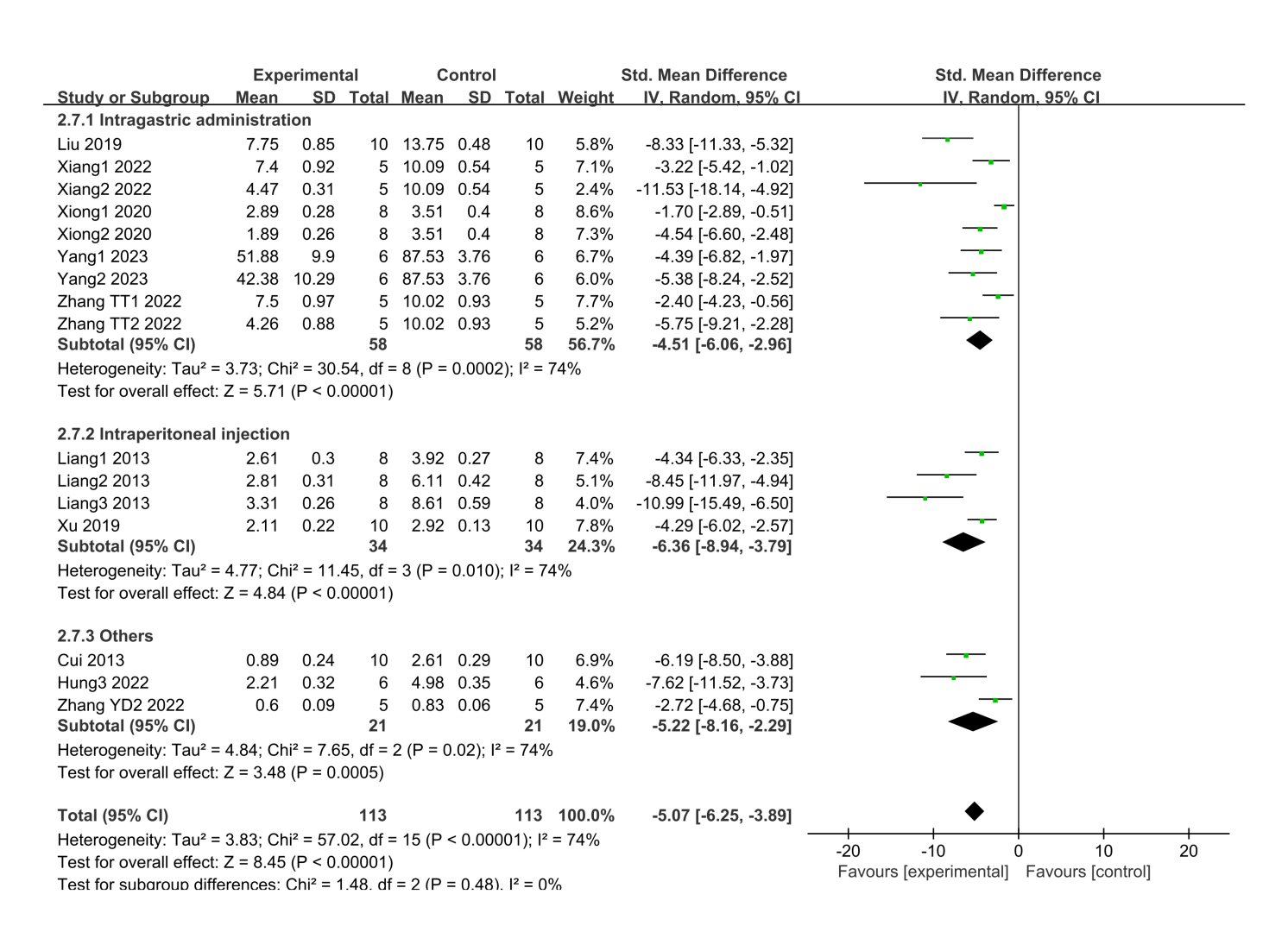
**Supplementary Figure 12.** Forest plot of subgroup analysis of the effect of SCFAs intervention on lung injury scores based on administration route.


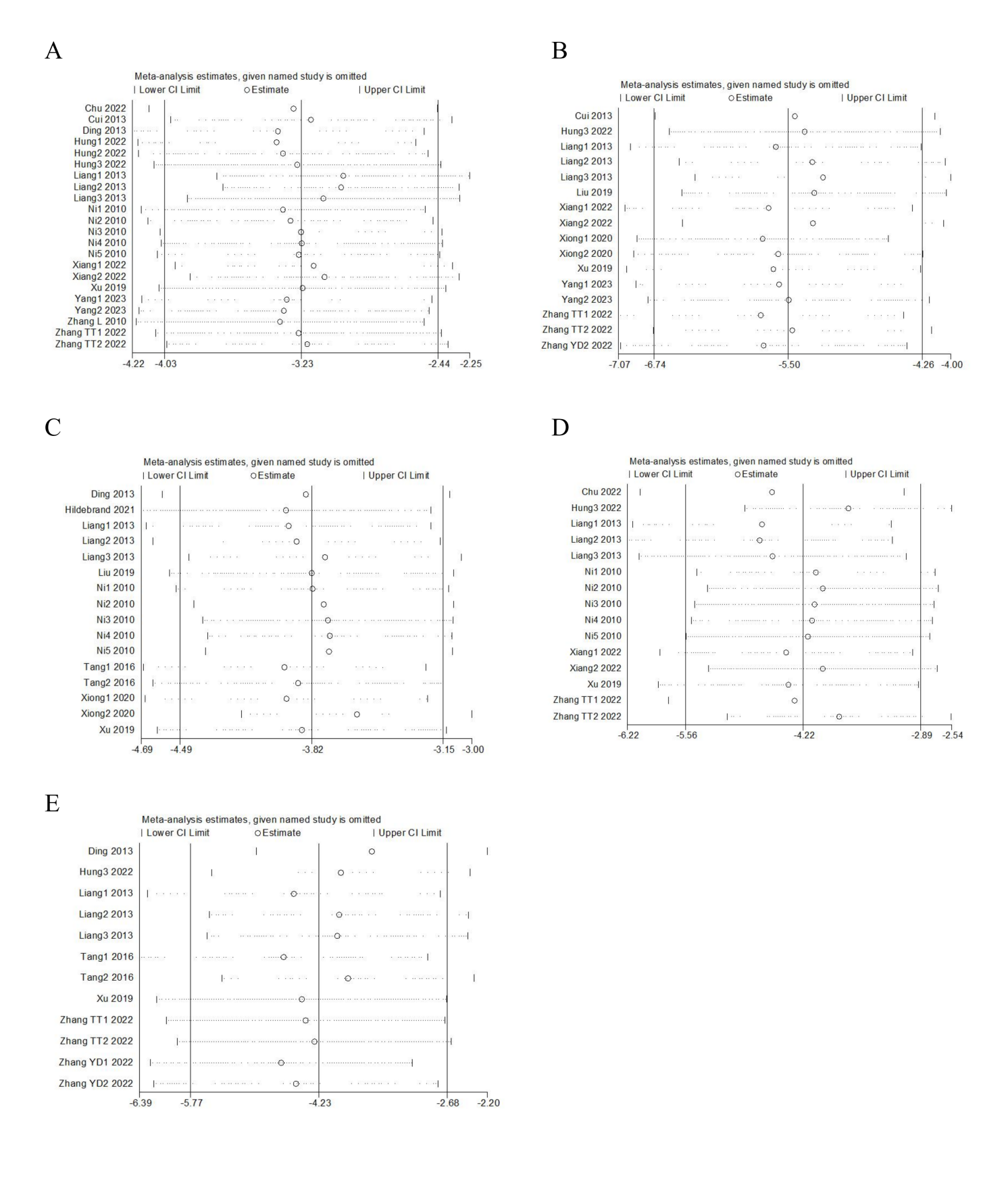
**Supplementary Figure 13.** Sensitivity analysis. A, Lung W/D ratio; B, Lung injury scores; C, MPO activity in lung tissue; D, TNF-α in BALF; E, MDA level in lung tissue.


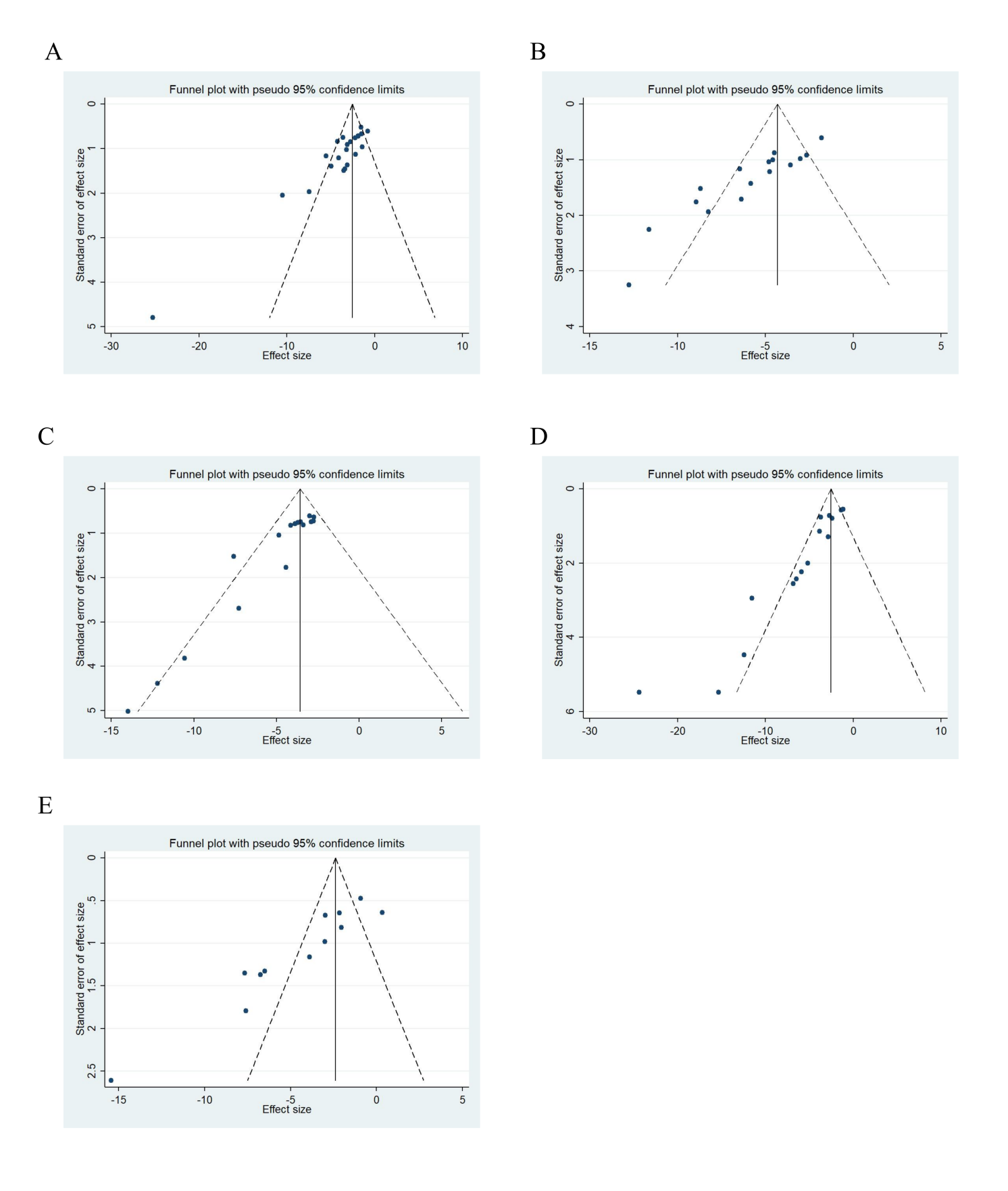
**Supplementary Figure 14.** Funnel plot for the assessment of publication bias. A, Lung W/D ratio; B, Lung injury scores; C, MPO activity in lung tissue; D, TNF-α in BALF; E, MDA level in lung tissue.


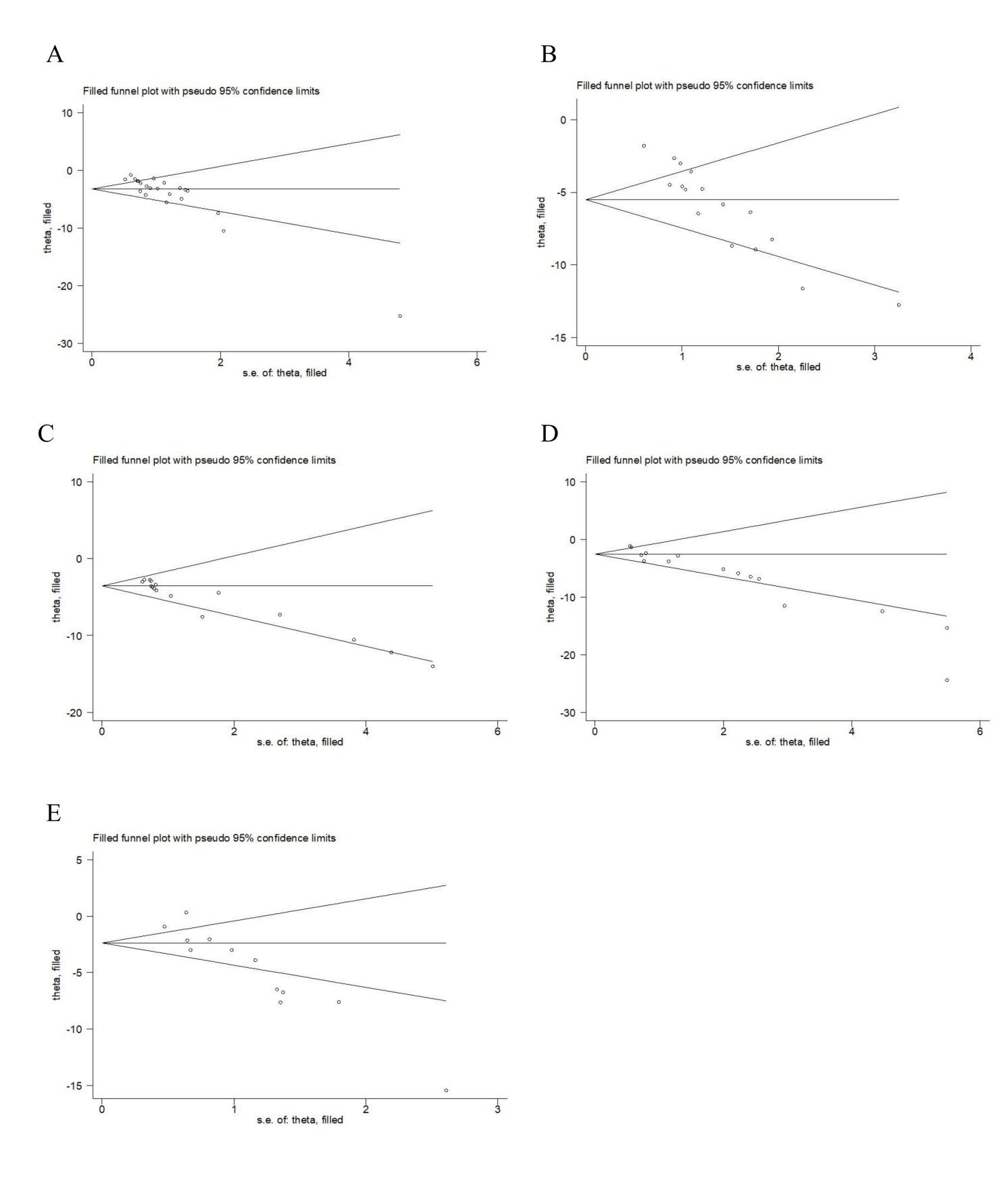
**Supplementary Figure 15.** Results of meta-analysis using the trim-and-fill method. A, Lung W/D ratio; B, Lung injury scores; C, MPO activity in lung tissue; D, TNF-α in BALF; E, MDA level in lung tissue.

## Supplementary Tables

**Supplementary Table 1.** Search Strategy

| Process | Keywords |
| --- | --- |
| # 1 | “acute lung injury” OR “acute respiratory distress syndrome” OR “ALI” OR “ARDS” |
| # 2 | “short chain fatty acid” OR “SCFA” OR “acetate” OR “propionate” OR “butyrate” |
| # 3 | # 1 AND # 2 |

**Supplementary Table 2.** The literature review summary table.

| **Author** | **Year** | **Title** | **Study aim** | **Region** | **Animal model (Gender)** | **Sample size (EG/CG)** | **Duration** | **Conclusion** |
| --- | --- | --- | --- | --- | --- | --- | --- | --- |
| Chu | 2022 | A High-Fiber Diet or Dietary Supplementation of Acetate Attenuate Hyperoxia-Induced Acute Lung Injury | To investigated the effects of acetate supplementation on the gut microbiota and hyperoxia-induced acute lung injury (HALI) in mice. | China | C57BL/6J mice (Male) | 6/6 | 3 weeks | Acetate supplementation reduced the severity of HALI through alterations in the gut microbiota to exert anti-inflammatory effects. |
| Cui | 2013 | The protective effect of NaB in rats of severe acute pancreatitis-associated lung injury | To investigate the protective effects of sodium butyrate on rats of severe acute pancreatitis-associated lung injury. | China | SD rats (Male) | 10/10 | 12 hours | Sodium butyrate can contribute to the apoptosis of rat lung tissue and alleviate the activation of neutrophil and lung injury. |
| Ding | 2014 | The protective effects of sodium butyrate on acute lung injury induced by lipopolysaccharide in rats | To investigate the protective effects of sodium butyrate on ALI induced by LPS in rats. | China | SD rats (Male) | 10/10 | 6 hours | Sodium butyrate exerted a protective effect by inhibiting neutrophil aggregation and cytokine production, as well as suppressing pulmonary lipid peroxidation and nitration. |
| Hildebrand | 2021 | Short-chain fatty acids improve inflamm-aging and acute lung injury in old mice | Short-chain fatty acids' function in the gut-lung axis in aging | Germany | C57BL/6 mice (Male) | 12/12 | 2 weeks | SCFAs played a beneficial role in the gut-lung axis of the aging organism by reducing pulmonary inflamm-aging and ameliorating enhanced severity of ALI in old mice. |
| Hung | 2023 | Acetate, a gut bacterial product, ameliorates ischemia-reperfusion induced acute lung injury in rats | To examine whether SCFAs have protective effects against IR-induced ALI and explore possible protective mechanisms. | China | SD rats (Male) | 6/6 | 60 minutes | Acetate may provide a novel adjuvant therapeutic approach for IR-induced ALI. |

**Supplementary Table 2 (Continued)**

| **Author** | **Year** | **Title** | **Study aim** | **Region** | **Animal model (Gender)** | **Sample size (EG/CG)** | **Duration** | **Conclusion** |
| --- | --- | --- | --- | --- | --- | --- | --- | --- |
| Li | 2018 | Sodium Butyrate alleviates LPS-induced Acute Lung Injuryin Mice via inhibiting HMGB1 release | To determine whether sodium butyrate can inhibit the release of inflammatory factors. | China | BALB/c mice (Male) | 10/10 | 24 hours | Sodium butyrate pretreatment can significantly reduce acute lung injury, manifested in significantly reducing lung dry-wet weight ratio, MPO activity and inflammatory cell infiltration. |
| Liang | 2013 | Sodium Butyrate Protects against Severe Burn-Induced Remote Acute Lung Injury in Rats | To investigate the effect of sodium butyrate on burn-induced lung injury. | China | SD rats (Female) | 8/8 | 12 hours, 24 hours, 48 hours | Sodium butyrate attenuated inflammatory responses, neutrophil infiltration, and oxidative stress in the lungs, and protects against remote ALI induced by severe burn. |
| Liu | 2019 | Sodium Butyrate Inhibits the Inflammation of LPS-induced Acute Lung Injury in Mice by Regulating the TLR4/NF-κB Signaling Pathway | To investigate the protective effects of sodium butyrate on LPS-induced ALI in mice and explore the potential mechanism of SB protection. | China | ICR mice (Female) | 10/10 | 12 hours | Sodium butyrate could inhibit inflammation in LPS-induced ALI. |
| Ni | 2010 | Histone deacetylase inhibitor, butyrate,attenuates lipopolysaccharide-induced acute lung injury in mice | To investigate the protective effect of butyrate, a HDAC inhibitor, on lipopolysaccharide (LPS)-induced acute lung injury (ALI) in mice. | China | BALB/c mice (Male) | 3/3 | 1 hour, 3 hours, 6 hours, 12 hours, 24 hours | Sodium butyrate had a protective effect on LPS-induced ALI, which may be related to its effect on suppression of inflammatory cytokines production. |

**Supplementary Table 2 (Continued)**

| **Author** | **Year** | **Title** | **Study aim** | **Region** | **Animal model (Gender)** | **Sample size (EG/CG)** | **Duration** | **Conclusion** |
| --- | --- | --- | --- | --- | --- | --- | --- | --- |
| Tang | 2015 | Protective effects of sodium butyrate acid on acute lung injury following intestinal ischemia － reperfusion in rats | To investigate the protective effects of sodium butyrate acid on ALI following intestinal ischemia-reperfusion in rats. | China | SD rats (Male) | 10/10 | 1 hour, 4 hours | Sodium butyrate could inhibit the increased inflammatory mediators and high microvascular permeability，and protect against ALI induced by I/R in rats. |
| Xiang | 2022 | Effects and mechanism of short chain fatty acids on lipopolysaccharide induced acute respiratory distress syndrome in rats | To investigate the effects and mechanism of SCFAs on LPS induced ARDS in rats. | China | SD rats (Male) | 5/5 | 7 days | SCFAs could regulate immunity and inflammation, and effectively improved LPS-induced ARDS in rats. |
| Xiong | 2020 | The changes and mechanisms of intestinal microbiota and Sodium butyrate in acute pancreatitis with acute respiratory distress syndrome | To explore the changes and mechanisms of intestinal microbiota and sodium butyrate in patients with ARDS | China | BALB/c mice (Male) | 8/8 | 7 days | High-dose sodium butyrate (500mg/kg) attenuated pancreas and lung injury in mice by inhibiting the HGMB1/NF-kB pathway. |
| Xu | 2019 | Intraperitoneal Injection of Acetate Protects Mice Against Lipopolysaccharide (LPS)‑Induced Acute Lung Injury Through Its Anti-Inflammatory and Anti-Oxidative Ability | To investigate the effect of acetate on LPS-induced ALI and explored its underlying mechanism. | China | C57BL/6J mice (Male) | 10/10 | 6 hours | Acetate exerted its protective effects via anti-inflammatory and anti-oxidant activities on LPS-induced ALI. |
| Yang | 2024 | Study on the regulation of macrophage polarisation by short-chain fatty acids in acute lung injury induced by sepsis in mice | To investigate the protective effect of SCFAs induced macrophage polarization on Sepsis Induced ALI in mice. | China | C57BL/6J mice (Male) | 6/6 | 24 hours | Sodium butyrate reduced lung tissue edema by decreasing the expression of lung tissue inflammatory factors, thereby alleviating ALI in sepsis. |

**Supplementary Table 2 (Continued)**

| **Author** | **Year** | **Title** | **Study aim** | **Region** | **Animal model (Gender)** | **Sample size (EG/CG)** | **Duration** | **Conclusion** |
| --- | --- | --- | --- | --- | --- | --- | --- | --- |
| Ying | 2020 | Sodium butyrate relieves lung ischemia-reperfusion injury by inhibiting NF-κB and JAK2/STAT3 signaling pathways | To investigate the efficacy and mechanism of sodium butyrate on lung ischemia-reperfusion injury. | China | C57BL/6J mice (Male) | NA | 1 week | Sodium butyrate relieved lung injury by inhibiting NF-κB and JAK2/STAT3 signaling pathways to reduce inflammation and oxidative stress levels in lung tissue of mice after IR. |
| Zhang L | 2011 | Histone Deacetylase Inhibitors Attenuate Acute Lung Injury During Cecal Ligation and Puncture-Induced Polymicrobial Sepsis | To investigate the effects of HDAC inhibitors on the expression of inflammation-related genes and lung injury during sepsis. | China | C57BL/6J mice (Male) | 6/6 | 18 hours | Sodium butyrate, based on modulating the key enzymes linked to acetylation modification, effectively attenuated intrapulmonary inflammatory response, thus significantly alleviating lung injury during sepsis. |
| Zhang TT | 2021 | Effects of sodium propionate on oxidative stress and inflammation in LPS induced acute lung injury rats | To investigate the effect of sodium propionate on oxidative stress and inflammation in rats with ALI induced by LPS. | China | SD rats (Male) | 5/5 | 7 days | Sodium propionate can reduce LPS-induced ALI in rats by regulating the Keap1/Nrf2 signaling pathway and inhibiting the activation of NF-κB signaling pathway, thereby reducing oxidative stress injury and inflammatory response. |
| Zhang YD | 2022 | Intestinal microbiota-derived propionic acid protects against zinc oxide nanoparticles- induced lung injury | To reveal an unknown bidirectional crosstalk mechanism between ALI and the perturbation of intestinal microbiota in the context of pulmonary exposure to ZnONPs, as well as the role of sodium propionate. | China | C57BL/6 mice (Male) | 5/5 | 21 days | Reveal a novel gut-lung axis mechanism in which intestinal microbiota and their derived metabolite propionic acid play protective roles against ZnONPs-induced ALI and suggest that FMT and supplementation with propionic acid are potential remedy strategies. |

Note. EG, experimental group; CG, control group; NaB, Natriumbutyrat, sodium butyrate; ALI, acute lung injury; LPS, lipopolysaccharides; SCFAs, short-chain fatty acids; I/R, ischemia/reperfusion; MPO, myeloperoxidase; ARDS, Acute Respiratory Distress Syndrome; CLP, cecal ligation and puncture; HMGB1, High Mobility Group Box 1; NF-kB, Nuclear Factor kappa-light-chain-enhancer of activated B cells; JAK2, Janus Kinase 2; STAT3, Signal Transducer and Activator of Transcription 3; NA, not applicable; Keap1, Kelch-like ECH-associated Protein 1; Nrf2, Nuclear Factor Erythroid 2–Related Factor 2; ZnONPs, Zinc Oxide Nanoparticles.

**Supplementary Table 3.** Risks of bias evaluated by the SYRCLE’s risk of bias tool.

| Author (Year) | 1 | 2 | 3 | 4 | 5 | 6 | 7 | 8 | 9 | 10 |
| --- | --- | --- | --- | --- | --- | --- | --- | --- | --- | --- |
| Chu  (2022) | Unclear | Unclear | Unclear | Yes | Unclear | Unclear | Unclear | Yes | Yes | Unclear |
| Cui  (2013) | Unclear | Unclear | Unclear | Yes | Unclear | Unclear | Unclear | Yes | Yes | Unclear |
| Ding  (2013) | Yes | Unclear | Unclear | Yes | Unclear | Unclear | Unclear | Yes | Yes | Unclear |
| Hildebrand (2021) | Unclear | Unclear | Unclear | Yes | Unclear | Unclear | Unclear | Yes | Yes | Unclear |
| Hung  (2022) | Unclear | Unclear | Unclear | Unclear | Unclear | Unclear | Unclear | Yes | Yes | Unclear |
| Li(2018) | Unclear | Unclear | Unclear | Yes | Unclear | Unclear | Unclear | No | Yes | Unclear |
| Liang  (2013) | Unclear | Unclear | Unclear | Yes | Unclear | Unclear | Unclear | Yes | Yes | Unclear |
| Liu(2019) | Unclear | Unclear | Unclear | Yes | Unclear | Unclear | Unclear | Yes | Yes | Unclear |
| Ni(2010) | Unclear | Unclear | Unclear | Unclear | Unclear | Unclear | Unclear | Yes | Yes | Unclear |
| Tang(2016) | Unclear | Unclear | Unclear | Unclear | Unclear | Unclear | Unclear | Yes | Yes | Unclear |
| Xiang  (2022) | Yes | Unclear | Unclear | Yes | Unclear | Unclear | Unclear | Yes | Yes | Unclear |
| Xiong  (2020) | Unclear | Unclear | Unclear | Unclear | Unclear | Unclear | Unclear | Yes | Yes | Unclear |
| Xu(2019) | Unclear | Unclear | Unclear | Yes | Unclear | Unclear | Unclear | Yes | Yes | Unclear |
| Yang(2023) | Yes | Unclear | Unclear | Yes | Unclear | Unclear | Unclear | Yes | Yes | Unclear |
| Ying(2021) | Unclear | Unclear | Unclear | Yes | Unclear | Unclear | Unclear | No | Yes | Unclear |
| Zhang L  (2010) | Unclear | Unclear | Unclear | Yes | Unclear | Unclear | Unclear | Yes | Yes | Unclear |
| Zhang TT  (2022) | Unclear | Unclear | Unclear | Yes | Unclear | Unclear | Unclear | Yes | Yes | Unclear |
| Zhang YD  (2022) | Unclear | Unclear | Unclear | Yes | Unclear | Unclear | Unclear | Yes | Yes | Unclear |

Note. Yes, indicates low risk of bias; no, indicates high risk of bias; unclear, indicates an unclear risk of bias.
